# Impact of Underlying Portal Hypertension on Severity and Course of Acute‐On‐Chronic Liver Failure

**DOI:** 10.1111/liv.70363

**Published:** 2025-09-23

**Authors:** Vlad Taru, Georg Kramer, Benedikt S. Hofer, Nina Dominik, Lorenz Balcar, Mathias Schneeweiss‐Gleixner, Bogdan Procopet, Michael Trauner, Mattias Mandorfer, Philipp Schwabl, Thomas Reiberger, Benedikt Simbrunner

**Affiliations:** ^1^ Division of Gastroenterology and Hepatology, Department of Medicine III Medical University of Vienna Vienna Austria; ^2^ Vienna Hepatic Hemodynamic Lab, Division of Gastroenterology and Hepatology, Department of Medicine III Medical University of Vienna Vienna Austria; ^3^ Iuliu Hatieganu University of Medicine and Pharmacy, 4th Dept. of Internal Medicine and “Octavian Fodor” Regional Institute of Gastroenterology and Hepatology, Hepatology Department Cluj‐Napoca Romania; ^4^ Christian‐Doppler Laboratory for Portal Hypertension and Liver Fibrosis Medical University of Vienna Vienna Austria; ^5^ Clinical Research Group MOTION Medical University of Vienna Vienna Austria; ^6^ CeMM Research Center for Molecular Medicine of the Austrian Academy of Sciences Vienna Austria

**Keywords:** acute decompensation, cirrhosis, hepatic venous pressure gradient, platelet, portal hypertension, systemic inflammation

## Abstract

**Background and Aims:**

The impact of portal hypertension (PH) during acute‐on‐chronic liver failure (ACLF) remains unclear. This study investigated the link between underlying PH severity, systemic inflammation (SI), and the course of ACLF.

**Methods:**

Consecutive patients with ACLF (*n* = 192) who met the EASL‐CLIF criteria were retrospectively included. PH severity (hepatic venous pressure gradient, HVPG; platelet count, PLT; and other clinical/radiologic PH surrogates) and SI (white blood cell count; C‐reactive protein [CRP], interleukin‐6) were assessed at the last pre‐ACLF visit, ACLF diagnosis (D0), and after 7 (D7), 28 (D28), and 90 (D90) days.

**Results:**

All patients had clinical/radiological signs of PH, and 91 (47%) patients developed ACLF grade 1, 62 (32%) ACLF‐2, and 39 (21%) ACLF‐3. Patients with different D0‐ACLF grades showed similar SI biomarker levels pre‐ACLF, whereas these increased significantly during ACLF. Median PLT decreased in parallel with the ACLF grade and from D0 (ACLF‐3:72; vs. ACLF‐2:81; vs. ACLF‐1:91 G/L; *p* = 0.094) to D7 (ACLF‐3:39 vs. ACLF‐2:64; vs. ACLF‐1:89 G/L; *p* < 0.001). In multivariable Cox regression models, D0‐PLT (aHR: 0.96 per 10 G/L [95% CI: 0.93–0.99], *p* = 0.015) independently predicted D28 mortality. A logistic regression model including sex, D0‐PLT, D0‐CRP, and CLIF‐C ACLF score predicted D28 mortality (AUROC: 0.79 [0.73–0.86]; *p* < 0.001) and outperformed (*p* = 0.036) the MELD‐Na score (AUROC: 0.71 [0.63–0.78]; *p* < 0.001).

**Conclusions:**

Although PH is a necessary condition for ACLF development, underlying PH severity does not confer a risk for higher ACLF severity but impacts survival after ACLF resolution. PLT emerged as a predictor of D28 mortality, independent of the CLIF‐C ACLF score.

**Summary**

- This study assessed how an increased blood pressure in the portal vein called ‘portal hypertension’ (PH) affects the risk of developing liver and other organ failure(s) on top of cirrhosis, a severe condition named acute‐on‐chronic liver failure (ACLF).
- We found that severe PH recorded in a patient before ACLF did not affect the severity of the ACLF episode or short‐term mortality.
- However, patients with severe PH had a higher chance of developing severe ACLF and a higher mortality risk in the long term.
- Patients with more severe ACLF experienced sudden drops in blood platelet levels.
- The drop in platelet levels 1 week after ACLF development can help doctors to assess the mortality risk of patients.

**Abbreviations**
ACLF: acute‐on‐chronic liver failure
AD: acute decompensation
ALD): alcohol‐related liver disease
CLIF‐C: chronic liver failure consortium
CRP: C‐reactive protein
CSPH: clinically significant portal hypertension
EASL: European Association for the Study of the Liver
HVPG: hepatic venous pressure gradient
IL‐6: interleukin‐6
LSM: liver stiffness measurements
MASLD: metabolic dysfunction‐associated steatotic liver disease
MELD: model for end‐stage liver disease
PH: portal hypertension
PLT: platelet count
SI: systemic inflammation
VWF: von Willebrand factor antigen
WBC: white blood cell (WBC) count

## 1 Introduction

The development of ascites, variceal bleeding and hepatic encephalopathy defines decompensated cirrhosis, and portal hypertension (PH) is the main driver of these complications [1]. If acute decompensation (AD) occurs in combination with specific patterns of dysfunction/failure of one or more extrahepatic organ systems, patients are diagnosed with acute‐on‐chronic liver failure (ACLF) [2], a syndrome with high short‐term mortality [2, 3] associated with a marked aggravation of systemic inflammation (SI).

Assessment of PH by hepatic venous pressure gradient (HVPG) is currently regarded as the gold standard, and clinically significant PH (CSPH), defined as an HVPG ≥ 10 mmHg, accurately identifies patients at risk for decompensation [4]. Platelet count (PLT) and liver stiffness (LSM) are established surrogates of PH severity in compensated cirrhosis, as evidenced by the validated ANTICIPATE model, and yielded a comparable prognostic utility for PH, as compared with HVPG [4, 5]. Bacterial translocation (BT) is considered a main contributor to SI, and SI may even occur in the early stages of cirrhosis [6]. PH severity affects the risk of BT and SI; PH treatment with non‐selective beta‐blockers may reduce BT and SI, which also translates into improved clinical outcomes for patients with cirrhosis [7, 8].

In turn, SI affects PH severity by mediating hepatic/sinusoidal vasoconstriction and aggravating systemic vasodilatation, and SI gradually increases with the development and higher grades of ascites [9].

Although there is an important body of evidence linking SI to the development of ACLF, the clinical significance of PH severity in ACLF has not been systematically explored, although CSPH is a prerequisite for hepatic decompensation, which is a *sine qua non* for ACLF, according to the EASL‐CLIF definition [10].

In this retrospective single‐centre study, we investigated the impact of PH severity on the grade and outcome of ACLF. Second, we aimed to evaluate how PH impacts the ACLF and whether surrogates of PH and SI improve the prognostication of patients with ACLF with regard to short‐term mortality.

## 2 Patients and Methods

### 2.1 Patient Selection and Study Design

We conducted a single‐centre retrospective study including patients who underwent hepatic vein catheterization for risk stratification of PH, who were admitted at Vienna General Hospital between November 2003 and November 2022 for AD, and met the European Association for the Study of the Liver–Chronic Liver Failure (EASL‐CLIF) diagnostic criteria for ACLF [2] (Figure S1).

Clinical, laboratory, hepatic haemodynamic, transient elastography, radiologic, and endoscopic parameters were collected from patients' medical records, as available, at the following time points: at the last pre‐ACLF visit, at ACLF diagnosis (D0), and at days 7 (D7), 28 (D28), and 90 (D90) after ACLF diagnosis, with strict intervals for each time point (see Supporting Information). Patients who had undergone orthotopic liver transplantation (LT), transjugular intrahepatic portosystemic shunt (TIPS) placement, or had been diagnosed with hepatocellular carcinoma (HCC) prior to ACLF diagnosis were excluded. Owing to the retrospective study design, these parameters were not consistently available. Missing values at different time points are indicated in Table S1.

### 2.2 Diagnostic Criteria of ACLF

The EASL‐CLIF criteria were used to diagnose ACLF [2]. Patients presenting with single kidney failure or any other organ failure in combination with either renal insufficiency (serum creatinine ≥ 1.5 mg/dL) or hepatic encephalopathy grade 1/2 were classified as ACLF grade 1, whereas those presenting with two or at least three organ failures were classified as ACLF‐2 and ‐3, respectively.

### 2.3 Evaluation of Portal Hypertension and Systemic Inflammation

The ISO‐certified Department of Laboratory Medicine at the Medical University of Vienna conducted routine laboratory tests and biomarker analyses, including von Willebrand factor antigen (VWF), interleukin (IL‐6) and C‐reactive protein (CRP), using commercially available methods approved for clinical use and blood sample analysis.

HVPG measurements were conducted at the Vienna Hepatic Hemodynamic Lab in accordance with standardised protocols as previously described [11]. Treatment with non‐selective beta‐blockers was suspended for at least 3 days before the measurement [12].

LSM was performed using FibroScan (Echosens, Paris, France) in accordance with established protocols, as previously described [13].

The presence and size of oesophageal varices (EVs) were evaluated during upper gastrointestinal endoscopy following national consensus guidelines [14, 15, 16], classifying them into three groups: no varices, small varices (< 5 mm), and large varices (> 5 mm).

Spleen size was assessed by abdominal ultrasound or computed tomography, and a craniocaudal diameter ≥ 13 cm (ultrasound) or > 10 cm (computed tomography) indicated splenomegaly [17].

Clinically significant portal hypertension (CSPH) was defined as an HVPG ≥ 10 mmHg, whereas additional clinical/imagistic signs reflecting PH were documented: (1) presence of collaterals on imaging, (2) presence of EVs, and (3) moderate to severe ascites [4].

### 2.4 Statistical Analysis

Statistical analyses were conducted using IBM SPSS Statistics v29 (IBM, Armonk, NY, USA) and R statistical software (R Foundation v4.3.3, Vienna, Austria). Continuous variables are presented as mean ± standard deviation (SD) or median with interquartile range (IQR—as absolute difference between Q25 and Q75), depending on the normality of the data assessed by normality plots and D'Agostino & Pearson and Shapiro–Wilk tests. Comparisons between independent/paired variables were conducted as appropriate using parametric or non‐parametric tests, according to the distribution. Survival times were analysed using Kaplan–Meier curves with log‐rank test for comparisons between groups, whereas the median follow‐up time was calculated using the Kaplan–Meier estimate of potential follow‐up [18]. Univariable and multivariable Cox proportional hazards models were used to assess the prediction of D28 mortality while censoring survivors at this time point. To build a prediction model for short‐term D28 mortality, a logistic regression model fitted with natural splines with three degrees of freedom for continuous variables was selected (see Supporting Information). Statistical significance was determined by a two‐sided *p*‐value below 0.05.

### 2.5 Ethical Aspects

This study adhered to the principles outlined in the 1964 Helsinki Declaration and its subsequent amendments and was approved by the local ethics committee of the Medical University of Vienna (EK1239/2023 and EK 1262/2017). The requirement for written informed consent was waived by the ethics committee of the Medical University of Vienna because of the retrospective study design.

## 3 Results

### 3.1 Patients' Characteristics

This study included 192 patients with a first episode of ACLF, of whom 91 (47.4%) had ACLF‐1, 62 (32.3%) ACLF‐2, and 39 (20.3%) ACLF‐3 (Table 1). The mean age at ACLF diagnosis was 57 ± 11.7 years and 121 (63%) patients had male sex. Alcohol‐related liver disease was the predominant aetiology recorded in 104 (54.2%) patients, followed by viral hepatitis in 41 (21.4%), cholestatic liver disease in 21 (10.9%), metabolic dysfunction‐associated steatotic liver disease in 15 (7.8%), and other aetiologies in 11 (5.7%), with no significant differences between ACLF grades (*p* = 0.834).

**TABLE 1:** Patient characteristics at ACLF diagnosis.

Patient characteristics	All	ACLF‐1	ACLF‐2	ACLF‐3	*p*
Patients (*n*, %)	192	91 (47)	62 (32)	39 (21)	
Age (years)	56.6 ± 11.7	59.5 ± 11.0	55.9 ± 11.1	51.0 ± 12.4^a^	**< 0.001**
Sex (male; *n*, %)	121 (63)	56 (62)	39 (63)	26 (67)	0.857
Aetiology (*n*, %)					0.834
ALD	104 (54)	52 (57)	35 (57)	17 (44)	
MASLD	15 (8)	6 (7)	5 (8)	4 (10)	
VIRAL	41 (21)	20 (22)	11 (18)	10 (26)	
CHOL	21 (11)	7 (8)	8 (13)	6 (15)	
OTHER	11 (6)	6 (7)	3 (5)	2 (5)	
Infection (*n*, %)	117 (61)	48 (53)	40 (65)	29 (74)	0.054
Type of OF (*n*, %)					
Liver	49 (26)	11 (12)	22 (36)	16 (41)	**< 0.001**
Kidney	127 (66)	57 (63)	42 (68)	28 (72)	**0.002**
Respiration	30 (16)	1 (1)	4 (7)	25 (64)	**< 0.001**
Circulation	49 (26)	1 (1)	14 (23)	34 (87)	**< 0.001**
Brain	77 (40)	19 (21)	27 (44)	31 (80)	**< 0.001**
Coagulation	30 (16)	3 (3)	15 (24)	12 (31)	**< 0.001**
PLT (G/L)	87 (91)	91 (93)	81 (96)	72 (86)	0.094
VWF (%)	420 (24)	420 (24)	420 (42)	398 (61)	0.130
WBC (G/L)	8.1 (6.9)	7.7 (6.9)	7.6 (6.1)	9.9 (8.1)^a^, ^b^	**0.029**
CRP (mg/dL)	2.9 (6.5)	2.7 (5.2)	2.6 (6.4)	6.5 (10.6)	0.124
IL‐6 (pg/mL)	4695 ± 12 659	389 ± 773	3459 ± 3417	8738 ± 18 257	**0.032**
Ammonia (μmol/L)	67.5 (67.3)	59.0 (57.7)	63.4 (73.2)	80.1 (74.5)	0.123
NSBB treatment (*n*, %)	116 (60.4)	54 (59.3)	38 (61.3)	24 (61.5)	0.959
MELD‐Na score	27.7 ± 5.8	25.6 ± 5.3	28.8 ± 5.3^a^	31.0 ± 5.9^a^	**< 0.001**
CLIF‐C OF score	9 (3)	8 (2)	10 (2)^a^	13 (3)^a^, ^b^	**< 0.001**
CLIF‐C ACLF score	48.0 ± 10.2	43.4 ± 7.0	47.4 ± 8.7^a^	59.6 ± 10.0^a^, ^b^	**< 0.001**
CLIF‐C AD score	62.5 ± 12.1	61.5 ± 9.7	61.4 ± 12.2	66.7 ± 16.1	0.056

*Note:* Statistical analysis: Metric variables are presented as median (IQR) or mean ± SD, depending on the normal distribution of variables. One‐way ANOVA and Kruskal–Wallis tests were used to compare parametric and non‐parametric variables, respectively, and adjusted for multiple testing using Tukey's or Dunn's corrections, respectively. *P* values in bold indicate statistical singificance (< 0.05).

Abbreviations: ACLF, acute‐on‐chronic liver failure; AD, acute decompensation; ALD, alcohol‐related liver disease; CHOL, cholestatic liver disease; CLIF‐C, Chronic Liver Failure Consortium; CRP, C‐reactive protein; HVPG, hepatic venous pressure gradient; IL‐6, interleukin 6; LSM, liver stiffness measurement; MASLD, metabolic dysfunction‐associated steatotic liver disease; MELD‐Na, model for end‐stage liver disease including sodium; OF, organ failure; PLT, platelet count; VWF, von Willebrand factor antigen; WBC, white blood cells.

^a^ *p* < 0.05 compared to ACLF‐1.

^b^ *p* < 0.05 compared to ACLF‐2.

Prior to the ACLF episode, clinical and radiological signs of portal hypertension, indicated by the presence of oesophageal varices, were reported in 138 (78.9% of known) patients, whereas splenomegaly was present in 141 (82% of known) and ascites in 144 (75%) patients (Table 2). LSM was available in 108 (56.3%) patients and had a median of 50.1 (42.7) kPa, with a value ≥ 25 kPa in 83.3% of patients. Laboratory values reported prior (less than 6 months) to the ACLF episode were available in 157 (81.8%) patients, with a median time to D0 of 69 (60–87) days and a median PLT of 101 G/L (< 150 G/L in 75.1%) (Table 2). A valid HVPG measurement was available in 186 (96.9%) patients, with a median time interval between HVPG measurement and ACLF diagnosis of 11 (1.1–30.2) months. The median HVPG was 19 mmHg (≥ 10 mmHg in 94.6%). Collectively, these surrogates indicated a PH prevalence of 100% in the study cohort. Non‐selective beta‐blocker (NSBB) treatment with either propranolol or carvedilol prior to ACLF was registered in 116 (60.4%). Patients with NSBB treatment showed more pronounced PH severity prior to ACLF compared to those without treatment, indicated by lower PLT (NSBB vs. No NSBB: 94 [60–134] vs. 116 [72–191] G/L, *p* = 0.036), higher LSM (NSBB vs. No NSBB: 63.3 [35.8–74.9] vs. 39.5 [27.1–65.8] kPa, *p* = 0.035) and higher HVPG (NSBB vs. No NSBB: 20 [17–23] vs. 18 [13–22], *p* = 0.006) (Figure S2).

**TABLE 2:** Patient characteristics prior to ACLF admission.

Patient characteristics	All	ACLF‐1	ACLF‐2	ACLF‐3	*p*
Varices (*n*, %)	138 (79)	73 (84)	42 (75)	23 (72)	0.204
Splenomegaly (*n*, %)	141 (82)	66 (80)	47 (87)	28 (80)	0.505
Ascites (*n*, %)	144 (79)	71 (83)	46 (77)	27 (75)	0.253
LSM (kPa)	50.1 (42.7)	53.6 (43.4)	58.2 (48.9)	42.9 (38.6)	0.774
HVPG (mmHg)	21 (8)	21 (7)	20 (9)	20 (8)	0.242
PLT (G/L)	101 (84)	101 (87)	101 (66)	110 (99)	0.908
VWF (%)	407 (111)	390 (99)	420 (152)	357 (139)	0.340
WBC (G/L)	6.0 (4.0)	5.8 (4.4)	6.0 (3.9)	6.2 (3.9)	0.575
CRP (mg/dL)	1.2 (2.5)	1.1 (3.0)	1.1 (2.3)	1.4 (2.5)	0.814
IL‐6 (pg/mL)	29.7 (43.8)	30.7 (58.0)	25.5 (21.4)	44.2 (47.3)	0.565
Ammonia (μmol/L)	54.5 (47.4)	45.3 (45.4)	65.2 (36.2)^a^	58.8 (53.7)	0.057
Child‐Pugh stage C (*n*, %)	67 (43)	28 (36)	22 (45)	17 (55)	0.321
MELD‐Na score (points)	20 ± 5	19 ± 6	20 ± 5	20 ± 5	0.140

*Note:* Statistical analysis: Metric variables are presented as median (IQR) or mean ± SD, depending on the normal distribution of variables. One‐way ANOVA and Kruskal–Wallis tests were used to compare parametric and non‐parametric variables, respectively, and adjusted for multiple testing using Tukey's or Dunn's corrections, respectively. *P* values in bold indicate statistical singificance (< 0.05).

Abbreviations: ACLF, acute‐on‐chronic liver failure; CRP, C‐reactive protein; HVPG, hepatic venous pressure gradient; IL‐6, interleukin 6; MELD‐Na, model for end‐stage liver disease including sodium; PLT, platelet count; VWF, von Willebrand factor antigen; WBC, white blood cells.

^a^ *p* < 0.05 compared to ACLF‐1.

^b^ *p* < 0.05 compared to ACLF‐2.

At the time of ACLF diagnosis (D0), infection was identified as the main precipitating factor in 117 (60.9%) patients. The most frequently observed organ failure was kidney failure in 127 (66.1%) patients, followed by brain failure in 77 (40.1%), liver and circulation failure in 49 (25.5%) each, and respiratory and coagulation failure in 30 (15.6%) each (Table 1).

### 3.2 Underlying Portal Hypertension and Systemic Inflammation Do Not Impact on ACLF Severity

**FIGURE 1:**
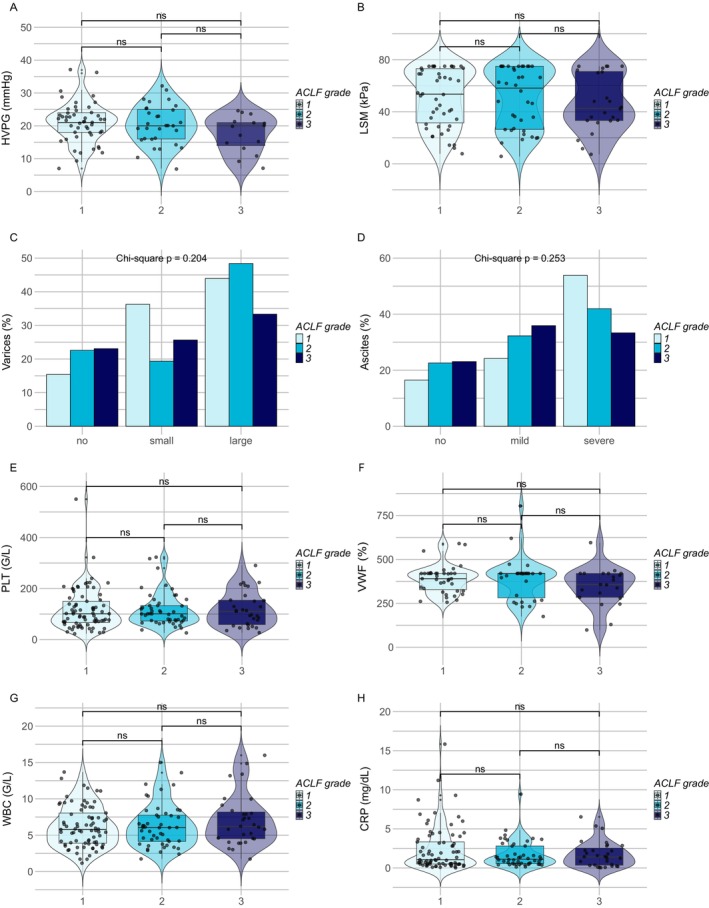
Pre‐ACLF severity of PH and SI according to ACLF grade at diagnosis. Ns, not significant; *p < 0.05; **p < 0.01; ***p < 0.01. Statistical analysis: Kruskal‐Wallis test was used for multiple‐groups comparisons of metric variables and adjusted for multiple testing using Dunn's correction. Chi‐square test was used for comparison of categorical variables. Abbreviations: CRP, C reactive protein; HVPG, hepatic venous pressure gradient; LSM, liver stiffness measurement; PLT, platelet count; VWF, von Willebrand factor antigen; WBC, white blood cell count.

To evaluate the impact of PH severity on ACLF, only patients with HVPG measurements within 1 year prior to ACLF were considered. In this subgroup of 95 (49.5%) patients, the median timespan between HVPG measurement and ACLF diagnosis was 1.1 (0.1–6.2) months. HVPG was similar across patients stratified by D0 ACLF grade (ACLF‐1: 21 (7) vs. ACLF‐2: 20 (9) vs. ACLF‐3: 20 (8), *p* = 0.242) (Figure 1A). When classifying patients according to the severity of PH, indicated by an HVPG ≥ 20 mmHg, 56 (58.9%) or < 20 mmHg, 39 (41.1%) patients, respectively, there were still no differences in ACLF grade at D0 (*p* = 0.674), but there were significantly more patients with ACLF‐3 in the HVPG ≥ 20 mmHg group at D7 (*p* = 0.046) (Table S2).

Similarly, other clinical surrogates of PH (LSM, presence of varices, splenomegaly, and grade of ascites) were not associated with D0 ACLF severity (Table 2, Figure 1B–D). Pre‐ACLF laboratory surrogates of PH (PLT and VWF) showed similar levels between the different D0 ACLF grades (Table 2). Although there was a trend towards lower PLT with higher ACLF grades on D0 (ACLF‐1: 91 (93) vs. ACLF‐2: 81 (96) vs. ACLF‐3: 72 (86) G/L, *p* = 0.094), this trend became significant on D7 (ACLF‐1: 89 (94) vs. ACLF‐2: 64 (47) vs. ACLF‐3: 39 (80) G/L, *p* < 0.001). The relative difference in PLT between D0 and D7 (delta PLT%) significantly increased with higher D0 ACLF grade (ACLF‐1: −15.9% vs. ACLF‐2: 23.5% vs. ACLF‐3: 45.1%, *p* = 0.039) (Table 3, Figure S3). There were no differences in distribution of ACLF grades according to the NSBB status, neither at D0 (ACLF‐1 vs. ACLF‐2 vs. ACLF‐3: 54 [59.3%] vs. 38 [61.3%] vs. 24 [61.5%], *p* = 0.959) nor at D7 (ACLF‐0 vs. ACLF‐1 vs. ACLF‐2 vs. ACLF‐3: 36 [67.9%] vs. 31 [59.6%] vs. 13 [54.2%] vs. 25 [59.5%], *p* = 0.656) (Figure S4).

**TABLE 3:** Patient characteristics and clinical course during and after ACLF.

Patient characteristics	All	ACLF‐1	ACLF‐2	ACLF‐3	*p*
At D7 (*n*, %)	172	86 (50)	59 (34)	27 (16)	
PLT (G/L)	70 (74)	89 (85)	64 (47)^a^	39 (80)^a^	**< 0.001**
Delta PLT (G/L)	−15 (52)	−15 (47)	−17 (61)	−22 (85)	0.206
Delta PLT (%)	−18 (51)	−15 (41)	−19 (48)	−31 (92)	**0.039**
WBC (G/L)	7.4 (6.7)	6.3 (6.0)	8.0 (5.3)	11.8 (12.5)^a^, ^b^	**0.003**
Delta WBC (G/L)	−0.2 (4.3)	−0.6 (3.2)	−0.1 (4.1)	2.6 (9.5)^a^	**0.016**
CRP (mg/dL)	3.0 (4.7)	2.2 (4.2)	4.2 (4.6)^a^	4.3 (4.7)^a^	**0.009**
Ammonia (μmol/L)	57.7 (49.6)	62.4 (55.3)	54.1 (32.5)	47.4 (56.8)	0.815
MELD‐Na score	26 ± 8	23 ± 8	28 ± 8^a^	28 ± 8^a^	**0.001**
CLIF‐C OF score	9 (5)	7 (3)	10 (4)^a^	13 (6)^a^, ^b^	**< 0.001**
CLIF‐C ACLF score	44.4 (18.8)	39.5 (13.0)	46.5 (18.3)^a^	59.9 (20.9)^a^, ^b^	**< 0.001**
CLIF‐C AD score	59.0 ± 12.7	58.0 ± 12.3	59.7 ± 12.9	60.3 ± 13.7	0.628
At D28 (*n*, %)	112	61 (54)	38 (34)	13 (12)	—
MELD‐Na score	23 ± 7	22 ± 7	22 ± 6	26 ± 8	0.221
CLIF‐C AD score	53.9 (10.5)	54.3 (9.3)	52.0 (8.9)	55.1 (10.5)	0.774
At D90 (*n*, %)	78	44 (56)	27 (35)	7 (9)	—
MELD‐Na score	19 (8)	19 (9)	19 (6)	19 (3)	0.925
CLIF‐C AD score	51.9 (10.4)	52.5 (9.5)	50.3 (11.3)	49.5 (6.5)	0.258
Liver‐related death (*n*, %)					
In hospital	90 (47)	29 (32)	30 (48)	31 (80)^a^, ^b^	**< 0.001**
D7 mortality	32 (17)	10 (11)	7 (11)	15 (39)^a^, ^b^	**< 0.001**
D28 mortality	77 (40)	26 (29)	23 (37)	28 (73)^a^, ^b^	**< 0.001**
D90 mortality	103 (55)	37 (42)	35 (59)	31 (82)^a^, ^b^	**< 0.001**
D180 mortality	115 (63)	44 (52)	38 (64)	33 (89)^a^, ^b^	**< 0.001**
1 year mortality	119 (66)	45 (54)	41 (71)^a^	33 (89)^a^, ^b^	**< 0.001**
Transplant‐free survival (median in days, 95% CI)	61 (33–130)	154 (80–611)	42 (28–194)	11 (5–22)^s^	**< 0.001**

*Note:* Statistical analysis: Metric variables are presented as median (IQR) or mean ± SD, depending on the normal distribution of variables. One‐way ANOVA and Kruskal‐Wallis tests were used to compare parametric and non‐parametric variables, respectively, and adjusted for multiple testing using Tukey's or Dunn's corrections, respectively. Survival rates were compared using log‐rank tests, and post hoc pairwise comparisons were adjusted for multiple testing using the Bonferroni correction. *P* values in bold indicate statistical singificance (< 0.05).

Abbreviations: ACLF, acute‐on‐chronic liver failure; AD, acute decompensation; CLIF‐C, Chronic Liver Failure Consortium; CRP, C‐reactive protein; MELD‐Na, model for end‐stage liver disease including sodium; OF, organ failure; PLT, platelet count; VWF, von Willebrand factor antigen; WBC, white blood cells.

^a^ *p* < 0.05 when compared to ACLF‐1.

^b^ *p* < 0.05 when compared to ACLF‐2.

Pre‐ACLF systemic inflammation markers (WBC, CRP and IL‐6) showed similar levels between the different ACLF grades (Table 2). At D0, the same markers increased significantly in parallel with the severity of ACLF and remained elevated during the ACLF episode on D7 (Table 3).

### 3.3 Dynamics of Portal Hypertension, Systemic Inflammation, and Disease Severity Prior, During and After ACLF Episode

**FIGURE 2:**
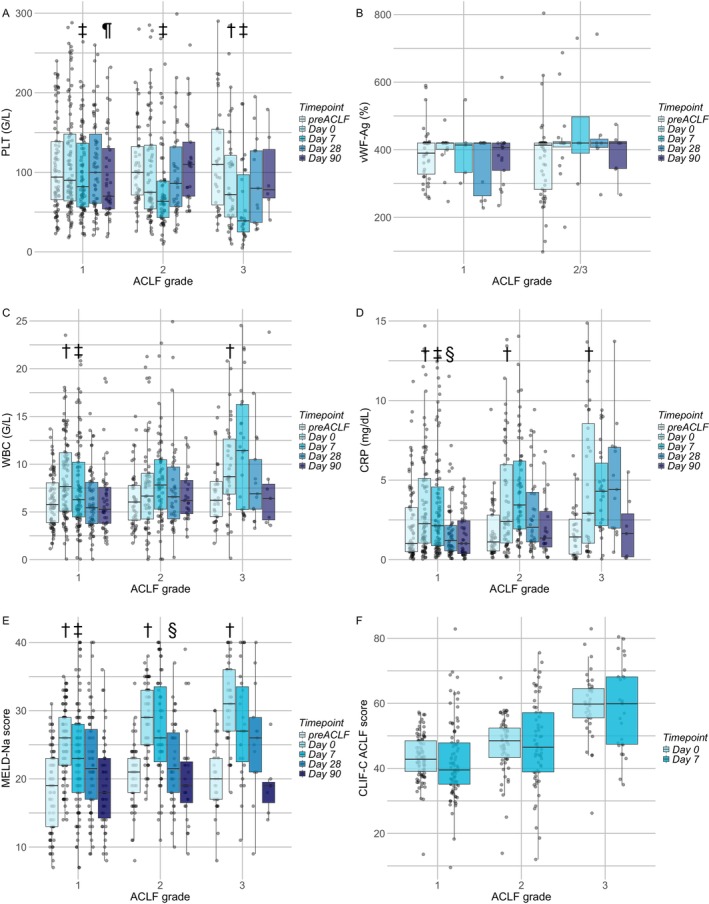
Dynamics of PH and SI surrogate biomarkers before, during and after ACLF. † *p* < 0.05 compared to pre‐ACLF; ‡ *p* < 0.05 compared to D0; § *p* < 0.05 compared to D7; ¶ *p* < 0.05 compared to D28. Statistical analysis: Paired Wilcoxon signed‐rank test was applied to compare the variables at different time points. Abbreviations: ACLF, acute‐on‐chronic liver failure; CLIF‐C ACLF, chronic liver failure acute‐on‐chronic liver failure score; CRP, C reactive protein; MELD, model for end‐stage liver disease; PLT, platelet count; VWF, von Willebrand factor antigen; WBC, white blood cell count.

Next, the dynamics of PH, SI markers and prognostic scores were analysed at different time points pre‐ACLF, during ACLF (D0 and D7) and after ACLF (D28 and D90) episode (Figure 2). The dynamics of PLT showed a progressive decrease during the ACLF episode, especially at D7 compared to D0 (all *p* < 0.05), and it was more evident in ACLF‐2 and ‐3 patients (Figure 2A). When comparing PLT levels in patients with and without confirmed infection on D0 and D7, there were no significant differences in the overall cohort or when stratified by ACLF grade at any of the time points (Figure S5). After the ACLF episode, there was a trend towards replenishing the PLT levels (Figure 2A).

Regarding SI surrogates, both WBC and CRP levels showed an acute increase at the time of ACLF diagnosis, which was consistent across all ACLF grades (Figure 2C,D). Although the levels of these markers decreased during ACLF episodes in patients with ACLF‐1, they remained elevated in patients with ACLF‐2 and ‐3. Similar to PLT, both WBC and CRP levels showed a trend towards the restoration of values post‐ACLF episodes. The MELD‐Na score was significantly higher at the time of ACLF diagnosis compared to pre‐ACLF across all ACLF grades (all *p* < 0.05) and remained high during the ACLF episode for ACLF‐2 and ‐3 patients (Figure 2E). The CLIF‐ACLF scores measured on D0 and D7 showed little variation between the two time points for all ACLF grades (Figure 2F).

Analysis of the PLT decrease (deltaPLT) potential mechanism revealed no significant association with SI progression (deltaCRP), but a significant association with coagulopathy (deltaFibrinogen) (Figure S6). Indeed, fibrinogen showed a similar trend as PLT during and after ACLF, with decreasing levels with higher ACLF grade and with progression from D0 to D7 (Figure S7).

### 3.4 Portal Hypertension and Systemic Inflammation Impact on ACLF Mortality

**FIGURE 3:**
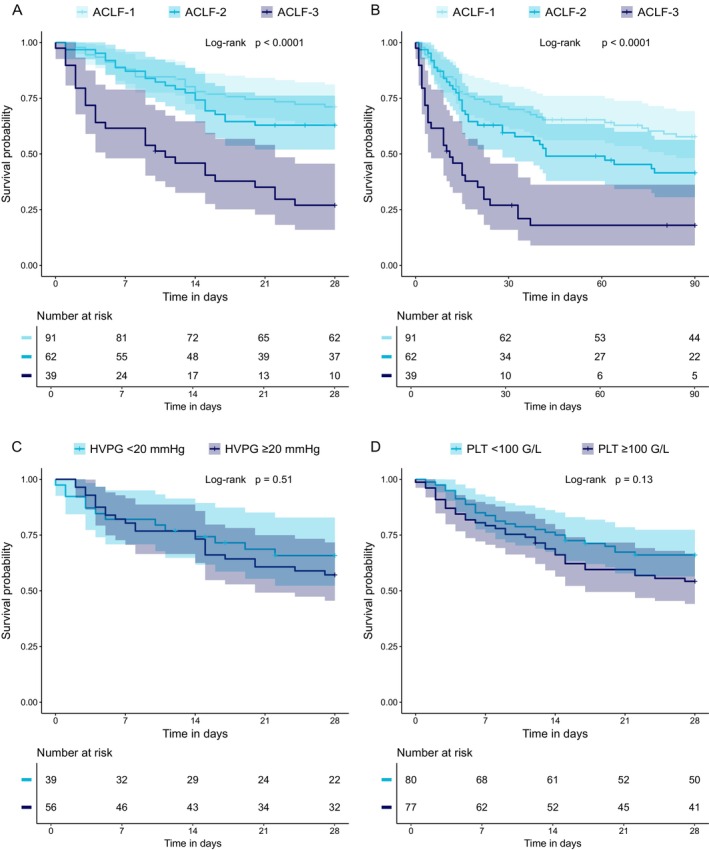
Short‐term survival according to ACLF grade and PH severity. Statistical analysis: Survival probabilities were plotted using Kaplan–Meier curves and compared between groups using the log‐rank test. Abbreviations: ACLF, acute‐on‐chronic liver failure; HVPG, hepatic venous pressure gradient; PLT, platelet count.

The median follow‐up time after ACLF diagnosis for the entire cohort was 70.2 (64.8) days. During follow‐up, 14 (7.3%) patients received TIPS, 15 (7.8%) underwent LT and 156 (81.3%) died. The median transplant‐free survival time following the first ACLF episode was 61 (95% CI: 33–130) days, with significantly shorter survival with higher ACLF grade (ACLF‐1: 154 (80–611) vs. ACLF‐2: 42 (28–194) vs. ACLF‐3: 11 (5–22) days, log‐rank *p* < 0.001) (Table 3). Short‐term mortality rates at 7, 28 and 90 days post‐ACLF episode were significantly higher with an increasing ACLF grade (log‐rank all *p* < 0.001) (Table 3, Figure 3A,B).

When assessing the impact of underlying PH on overall mortality, patients within the HVPG ≥ 20 mmHg group showed a lower median transplant‐free survival compared to the HVPG < 20 mmHg group (HVPG ≥ 20 mmHg: 2.5 (0.7–16.9) vs. HVPG < 20 mmHg: 6.6 (1.3–50.1) months, log‐rank *p* = 0.035). Interestingly, in‐hospital and short‐term mortality rates (at D7, D28, and D90) following the first ACLF episode were non‐significantly different between the two groups (log‐rank all *p* > 0.05) (Figure 3C, Table S2). Prior treatment with NSBB did not impact on ACLF day‐90 mortality (NSBB vs. No NSBB: 51.7% vs. 58.9%, log‐rank *p* = 0.490) (Figure S4).

When stratifying patients on the basis of median D0‐PLT (≥ 90 G/L, *n* = 94 vs. < 90 G/L, *n* = 98), patients with PLT < 90 G/L had significantly higher D90 mortality than those with PLT ≥ 90 G/L (64% [53–73] vs. 46% [34–55], log‐rank *p* = 0.01) (Figure 3D).

To identify which parameters were associated with D28 survival after an ACLF episode, we performed a univariable Cox proportional hazard regression analysis (Table 4). No parameter reflecting PH or SI assessed prior to the ACLF episode was associated with the D28 survival. When analysing the parameters assessed at the time of ACLF diagnosis, PLT, WBC, MELD‐Na, CLIF‐C AD and CLIF‐C ACLF scores were significantly associated with D28 survival. Additionally, D0‐PLT was associated with D90 but not with D7 survival (Table S3). In the multivariable Cox regression analysis, including sex, PLT, CRP, and CLIF‐C ACLF score on D0, PLT (aHR 0.96 per 10 G/L; 95% CI 0.93–0.99; *p* = 0.015) and CLIF‐C ACLF (aHR 1.08 per point; 95% CI 1.06–1.11; *p* < 0.001) remained independently associated with short‐term survival (Table 4). Next, we assessed the association between parameters measured at D7 and their change between D0 and D7 (delta) (Table S4). Consistent with previous results, delta PLT (aHR 0.987 per 10 G/L; 95% CI 0.979–994; *p* < 0.001) and delta CLIF‐C ACLF (aHR 1.15 per point; 95% CI 1.11–1.18; *p* < 0.001) were independently associated with short‐term survival.

**TABLE 4:** Predictors of ACLF short‐term (28‐day) mortality.

Covariates	Univariable analysis			Multivariable analysis (last step)		
	HR	95% CI	*p*	aHR	95% CI	*p*
Age (per year)	0.99	0.97–1.01	0.559			
Sex (male)	1.04	0.65–1.65	0.881	0.90	0.56–1.43	0.649
Prior to ACLF						
PLT (per 10 G/L)	0.97	0.93–1.01	0.122			
VWF (per 10%)	0.99	0.96–1.02	0.465			
HVPG (per mmHg)	1.00	0.95–1.06	0.869			
WBC (per G/L)	1.06	0.98–1.15	0.167			
CRP (per mg/dL)	0.94	0.84–1.07	0.353			
Child‐Pugh score (per point)	1.12	0.99–1.27	0.085			
MELD‐Na score (per point)	1.02	0.98–1.07	0.361			
CLIF‐C AD score (per point)	1.01	0.98–1.05	0.381			
At ACLF diagnosis (D0)						
PLT (per 10 G/L)	0.96	0.93–0.99	**0.027**	0.96	0.93–0.99	**0.015**
WBC (per G/L)	1.04	1.02–1.06	**< 0.001**			
CRP (per mg/dL)	1.04	1.01–1.07	**0.014**	1.03	0.99–1.06	0.099
Child‐Pugh score (per point)	1.44	1.27–1.62	**< 0.001**			
MELD‐Na score (per point)	1.13	1.01–1.18	**< 0.001**			
CLIF‐C AD score (per point)	1.07	1.05–1.09	**< 0.001**			
CLIF‐C ACLF score (per point)	1.08	1.06–1.11	**< 0.001**	1.08	1.06–1.11	**< 0.001**

*Note:* Statistical analysis: univariable and multivariable (backward stepwise method) Cox proportional hazard models were used to determine the prognostic value of metric variables towards mortality risk at D28. *P* values in bold indicate statistical singificance (< 0.05).

Abbreviations: CI, confidence interval; CLIF‐C AD/ACLF, chronic liver failure acute decompensation/acute‐on‐chronic liver failure score; CRP, C reactive protein; HR, hazard ratio; HVPG, hepatic venous pressure gradient; MELD, model for end‐stage liver disease; PLT, platelet count; VWF, von Willebrand factor antigen; WBC, white blood cell count.

### 3.5 Assessment of a Prediction Model Including CLIF‐C ACLF Score and PLT at D0

Subsequently, we assessed the value of adding PLT to the CLIF‐C ACLF score at D0 to improve the performance of the score in predicting short‐term survival in patients with ACLF. To this end, a multivariable logistic regression model was built, which included sex, log_2_(PLT), log_2_(CRP) and CLIF‐C ACLF score assessed on D0 as covariates and death on D28 as the outcome variable (Figure S8). In the bootstrap validation analysis, the prediction model achieved a training and testing C‐index of 0.83 and 0.77, respectively, with a calibration slope = 0.73 (Table S5 and Figure S9). More detailed model diagnostics are presented in the Supporting Information. Next, the performances of MELD‐Na (AUROC: 0.71 (0.63–0.78), *p* < 0.001), CLIF‐C ACLF (AUROC: 0.76 (0.70–0.83), *p* < 0.001), and the new model (AUROC: 0.79 (0.73–0.86), *p* < 0.001) were assessed in the entire cohort, with the new model performing significantly better than the MELD‐Na score (*p* = 0.036) and numerically outperforming the CLIF‐C ACLF score (*p* = 0.151) (Figure S10).

## 4 Discussion

This study assessed the effect of underlying PH severity on the clinical outcomes of patients with ACLF. Importantly, PH severity was characterised by various clinical, radiological, and laboratory assessments and by the diagnostic reference standard HVPG prior to ACLF development. Although pre‐ACLF PH severity did not impact on ACLF grade at diagnosis and short‐term survival, significantly more patients with HVPG ≥ 20 mmHg progressed to higher ACLF grades during admission, and survivors of the ACLF episode had worse long‐term clinical outcomes, compared to patients with HVPG < 20 mmHg. Surprisingly, PLT dynamic assessed at different time points during the ACLF course, which may reflect both PH/liver failure and SI severity, was an independent predictor of short‐term mortality at D28 after ACLF.

Although SI is an established driver of disease progression from non‐AD to AD and ACLF in cirrhosis patients [2, 3], the impact of PH after decompensation development is less clear [19]. Interestingly, the ‘PREDICT’ study, including more than 1000 patients with AD reported a higher prevalence of gastrointestinal haemorrhage and circulatory dysfunction in patients without an ACLF episode during the 90‐day follow‐up, as compared with those who developed ACLF (pre‐ACLF) during the same period [3].

A study from our centre previously showed that PH assessed by HVPG was similar between patients with different patterns of AD, including stable decompensation (SDC), unstable decompensation (UDC) and pre‐ACLF [20]. Similarly, in the current study, HVPG was not associated with ACLF grade at diagnosis or with short‐term mortality at D28 following ACLF. However, significantly more patients with severe PH, reflected by HVPG ≥ 20 mmHg, progressed to ACLF‐3 by D7 compared to those with HVPG < 20 mmHg. In addition, among the patients who survived the ACLF episode, those with more pronounced PH (HVPG ≥ 20 mmHg) had worse overall transplant‐free survival. Together, these findings indicate that the underlying severity of PH is not the main factor determining AD/ACLF severity but may impact the course and long‐term outcomes. Our study could not capture acute rises of PH that might occur in ACLF, as reported in patients with acute variceal bleeding [21, 22] or severe alcohol‐related hepatitis (SAH) [23, 24]. In acute variceal bleeding, continuous HVPG measurement can accurately predict the course of bleeding and survival, and a value above 20 mmHg is associated with poor clinical outcomes, including failure to control bleeding and in‐hospital mortality [22]. Acute changes in PH are also more pronounced in SAH patients with ACLF compared to AD and are independently associated with D90 mortality [24].

Intriguingly, in our study, SI biomarkers assessed prior to ACLF were also similar between patients with different ACLF severity grades at diagnosis. Whether there was different SI severity in patients prior to developing ACLF was not specifically addressed in the PREDICT cohort, where markers of SI assessed prior to ACLF were not stratified by subsequent ACLF grade. Notably, we found an increase in SI biomarkers, including WBC count and CRP level, with the development and severity of ACLF, as previously shown [2, 3]. Consistent with previous reports, we could show that SI surrogate markers decrease early at D7 and resume to values prior to ACLF at D28 in patients with ACLF‐1, whereas they continued to increase at D7 and remained elevated at D28 in ACLF‐2 and ‐3.

Thrombocytopenia, defined as PLT < 150 G/L, is common in patients admitted to intensive care units and is a parameter of the Sepsis‐related Organ Failure Assessment (SOFA) score, as it represents an independent predictor of death among critically ill patients [25, 26]. In patients with sepsis, thrombocytopenia is common and is associated with an increased risk of haemorrhage, renal injury and prolonged stay in the intensive care unit, and independently predicts worse outcomes [27]. Importantly, thrombopoiesis is stimulated in sepsis patients, as indicated by an elevated reticulated platelet percentage and circulating thrombopoietin (TPO) [28]. Nevertheless, thrombocytopenic pathomechanisms, such as platelet consumption via immune‐mediated microvascular thrombosis, autoantibodies targeted against platelets, sequestration in the liver and spleen, or consumptive coagulopathy presenting as disseminated intravascular coagulation (DIC), surpass platelet production [27].

In patients with cirrhosis, thrombocytopenia reflects the severity of liver failure and PH and is independently associated with the risk of portal vein thrombosis (PVT) [29] and PH‐related bleeding events [30]. In contrast to sepsis, cirrhosis is characterised by a progressive decrease in platelet production, which reflects PH severity. PH may induce hypersplenism and increase platelet turnover; however, the significance of this mechanism has been reconsidered over the past two decades, as interventional or surgical treatments targeting PH have not consistently or durably resolved thrombocytopenia [31]. An important unresolved scientific question is whether increased PLT activation upon stimulation contributes to thrombotic events in patients with cirrhosis and thrombocytopenia [32, 33].

In the context of AD and ACLF, limited data are available on PLT dynamics, function or prognostic value [34]. In line with previous reports [2, 3], our results indicated that PLT levels decreased in patients who developed ACLF and became significantly lower with increasing ACLF severity. Furthermore, the rapid decrease in PLT during ACLF was not associated with infections or SI in this study, indicating an independent pathomechanism for PLT consumption. Additionally, PLT acute decrease may be related to coagulopathy (i.e., DIC), rather than with an acute increase in PH severity, as indicated by the positive association with fibrinogen levels in our study. Importantly, we showed that in survivors of the ACLF episode, PLT restored at D28 in ACLF‐1 and ACLF‐2 patients but remains low in ACLF‐3. Additionally, PLT measured at ACLF diagnosis served as a good prognostic marker for both short‐ and long‐term transplant‐free survival. A PLT value of < 90 G/L at ACLF diagnosis was associated with excess mortality on day 90. In a multivariable model including sex, PLT, CRP and CLIF‐C ACLF score at ACLF diagnosis, PLT and CLIF‐C ACLF scores were independently associated with D28 mortality. The logistic regression model to predict D28 mortality, adjusted for sex, PLT, CRP and CLIF‐C ACLF score, showed good calibration and risk discrimination, significantly outperforming MELD‐Na. Although the addition of PLT to the already established CLIF‐C ACLF score numerically improved its prognostic performance, this newly developed model requires further validation in an external cohort of patients with ACLF.

Our study also has limitations: (i) the retrospective design, which impacts the quality and availability of parameters; (ii) inclusion of consecutive ACLF patients who underwent hepatic vein catheterisation. Although these inclusion criteria may limit the generalisability of our results, ACLF severity and survival estimates observed in our cohort match those reported in two large European multicentre prospective studies of AD/ACLF patients [2, 3], supporting the robustness of our data; (iii) analysis of several consecutive time points during the course of ACLF, which is characterised by high short‐term mortality, resulted in capturing data only for survivors at the respective time points; and (iv) the study covered a long period of 20 years, which is subject to changes in patient management of diseases and potential variance in liver disease epidemiology and characteristics. Although sensitivity analysis using multiple imputation methods supported the robustness of our findings, residual bias from informative missingness cannot be fully excluded. Future studies with prospective, protocol‐driven lab collection and follow‐up are warranted to validate our findings.

In conclusion, this study investigated the clinical outcomes of a large cohort of well‐characterised ACLF patients with regard to underlying PH and SI severity. Although pre‐ACLF PH did not affect the subsequent ACLF grade and short‐term survival, patients with more pronounced PH progressed to higher ACLF grades by D7 and had worse long‐term clinical outcomes. A significant dynamic in the PLT count during ACLF was observed, with particularly pronounced thrombocytopenia in ACLF grades 2 and 3. Notably, PLT count held significant prognostic value on top of the established CLIF‐C ACLF score to predict short‐term mortality in ACLF patients. Further studies on the pathomechanisms involved in the sudden decrease in PLT and to independently validate its prognostic value in patients with higher‐grade ACLF are warranted.

## Author Contributions

The authors contributed as follows: study conceptualization: V.T., G.K., B.S., and T.R.; data curation: all authors; formal analysis: V.T., B.S., and T.R.; interpretation: all authors; and supervision: T.R. and B.S. V.T. and G.K. drafted the original manuscript, which was critically reviewed and edited by all other authors. Funding acquisition was secured by T.R. V.T. and G.K. contributed equally and share the positions of the first author.

## Ethics Statement

This study adhered to the principles outlined in the 1964 Helsinki Declaration and its subsequent amendments and was approved by the local ethics committee of the Medical University of Vienna (EK1239/2023 and EK 1262/2017).

## Consent

The requirement for written informed consent was waived by the ethics committee of the Medical University of Vienna because of the retrospective study design.

## Conflicts of Interest

Vlad Taru (V.T.) was funded by the Christian Doppler Research Association and Boehringer Ingelheim RCV GmbH & Co KG (CD10271603), Romanian Ministry of Education (HG 118/2023), and Romanian Ministry of Research, Innovation and Digitalization (PNRR/2022/C9/MCID/I8). Benedikt Silvester Hofer (B.S.H.) received travel support by Ipsen. Lorenz Balcar (L.B.) received speaker fees from Chiesi, and Gilead. Bogdan Procopet (B.P.) received speaking honoraria from Abbvie, Echosens, consulting/advisory board fees from Boehringer‐Ingelheim, and travel support from Abbvie, Alfasigma. Michael Trauner (M.T.) has received research grants from Albireo, Alnylam, Cymabay, Falk, Genentech, Gilead, Intercept, MSD, Takeda, and Ultragenyx, and travel grants from Abbvie, Falk, Gilead, Intercept, and Jannsen. He further advised Abbvie, Agomab, Albireo, Agomab, BiomX, Boehringer Ingelheim, Chemomab Falk Pharma GmbH, Genfit, Gilead, Hightide, Intercept, Ipsen, Janssen, MSD, Novartis, Phenex, Pliant, Regulus, Siemens, and Shire, and has served as a speaker for Albireo, BMS, Boehringer Ingelheim, Falk, Gilead, Intercept, Ipsen, Madrigal, and MSD. He is a co‐inventor (service invention) of patents for the medical use of norUDCA (nor‐ursodeoxycholic acid/norucholic acid) filed by the Medical Universities of Graz and Vienna. Mattias Mandorfer (M.M.) received grants from Echosens; served as a speaker and/or consultant and/or advisory board member for AbbVie, Collective Acumen, Echosens, Gilead, Ipsen, Takeda, and W. L. Gore & Associates; and received travel support from AbbVie and Gilead. Philipp Schwabl (P.S.) received consulting/advisory board fees from PharmaIN and travel support from Dr. Falk Pharma. Thomas Reiberger (T.R.) received grant support from Abbvie, Boehringer‐Ingelheim, Gilead, Gore, Intercept, MSD, Myr Pharmaceuticals, Philips Healthcare, Pliant, and Siemens; speaking honoraria from Abbvie, Gilead, Gore, Intercept, Roche, MSD; consulting/advisory board fees from Abbvie, Bayer, Boehringer‐Ingelheim, Gilead, Intercept, MSD, Siemens; and travel support from Abbvie, Boehringer‐Ingelheim, Gilead, and Roche. Benedikt Simbrunner (B.S.) received travel support from AbbVie, Gilead, and Falk.

## Supporting information


**Data S1:** liv70363‐sup‐0001‐supinfo.docx.

## Data Availability

Data, analytical methods, and study materials are available upon reasonable request to the corresponding author.
