# Supplementary material for: Impact of Underlying Portal Hypertension on Severity and Course of Acute‐On‐Chronic Liver Failure
Source: Liver Int. 2025 Sep 23;45(10):e70363. doi: 10.1111/liv.70363 (PMC12456108; doi:10.1111/liv.70363)
Supplement: Supplementary file 1 — Data S1: liv70363‐sup‐0001‐supinfo.docx. [file LIV-45-0-s001.docx]

**SUPPLEMENTARY MATERIAL**

**Title:** Impact of underlying portal hypertension on severity and course of acute-on-chronic liver failure

Vlad Taru, MD^1,2,3,4†^

Georg Kramer, MD^1,2,4,5†^

Benedikt Silvester Hofer, MD^1,2,4,5,6^

Nina Dominik, MD^1,2,5^

Lorenz Balcar, MD^1,2,5^

Mathias Schneeweiss-Gleixner, MD PhD^1,2^

Bogdan Procopet, MD PhD^3^

Michael Trauner, MD^1,5^

Mattias Mandorfer, MD PhD^1,2,5^

Philipp Schwabl, MD PhD^1,2,4,5,6^

Thomas Reiberger, MD^1,2,4,5,6^*

Benedikt Simbrunner, MD PhD^1,2,4,5,6^

1. Division of Gastroenterology and Hepatology, Department of Medicine III, Medical University of Vienna, Vienna, Austria
2. Vienna Hepatic Hemodynamic Lab, Division of Gastroenterology and Hepatology, Department of Medicine III, Medical University of Vienna, Vienna, Austria
3. Iuliu Hatieganu University of Medicine and Pharmacy, 4^th^ Dept. of Internal Medicine and "Octavian Fodor” Regional Institute of Gastroenterology and Hepatology, Hepatology Department, Cluj-Napoca, Romania, Cluj-Napoca, Romania
4. Christian-Doppler Laboratory for Portal Hypertension and Liver Fibrosis, Medical University of Vienna, Vienna, Austria
5. Clinical Research Group MOTION, Medical University of Vienna, Vienna, Austria
6. CeMM Research Center for Molecular Medicine of the Austrian Academy of Sciences, Vienna, Austria

(†) These authors contributed equally to this manuscript.

(*) Corresponding author.

**Table of contents**

[**Supplementary methods** 3](#_Toc175055944)

[*Timepoints and intervals for data collection* 3](#_Toc175055945)

[*Statistical analysis – group comparison and prediction model construction* 3](#_Toc175055946)

[**Supplementary figures** 5](#_Toc175055947)

[**Supplementary tables** 11](#_Toc175055948)

# **Supplementary methods**

## *Timepoints and intervals for data collection*

When collecting data at different timepoints, narrow intervals were allowed to ensure a greater availability of parameters, as follows: last pre-ACLF visit – laboratory data between 1 to 3 months, and HVPG measurement no older than 1 year prior to the ACLF diagnosis; D0 – between 3 days prior and 3 days after ACLF diagnosis; D7 – between 4 and 10 days after ACLF diagnosis; D28 - between 21 and 35 days after ACLF diagnosis and D90 – between 60 and 120 days after ACLF diagnosis.

## *Statistical analysis – group comparison and prediction model construction*

For comparisons between two groups, Student t-test or Mann–Whitney U test was used for metric variables with normal and non-normal distribution, respectively. One-way ANOVA and Kruskal-Wallis tests were applied for multi-group comparisons of metric variables with normal and non-normal distribution, respectively, and P values were corrected for multiple group comparisons using Tukey’s and Dunn’s corrections, respectively. Paired metric variables were compared by Wilcoxon signed-rank paired test. Categorical variables were analyzed using the Chi-square test or Fisher’s Exact test, as appropriate.

To build a prediction model for early death at 28-day after ACLF diagnosis, we selected sex, PLT, CRP and CLIF-C ACLF score as covariates, based on the clinical relevance of these parameters and to avoid collinearity with the variables included in the validated CLIF-C ACLF score. First, we performed initial data analysis for the selected variables and base 2 logarithmic transformation for the right skewed ones. A multivariable logistic regression model was used including sex, log_2_(PLT), log_2_(CRP) and CLIF-C ACLF score as independent variables and death at D28 as the outcome variable. For continuous variables we fitted the model with restricted cubic splines with three degrees of freedom. We performed a bootstrap (n=100) validation using the *rms* package in R statistical software (The R Foundation, Vienna, Austria). The diagnosis of the model included the C-indexes, intercept, calibration slope, maximum absolute error in predicted probabilities (Emax), discrimination coefficient and Brier score to measure the accuracy of probabilistic predictors. Finally, we compared the model prognostic performance with previously established scores using the receiver operating characteristics (ROC) analysis, the area under the curve (AUC) and DeLong’s test to compare the AUCs.

# **Supplementary figures**

**Supplementary Figure-S1. Patient selection flow chart.**


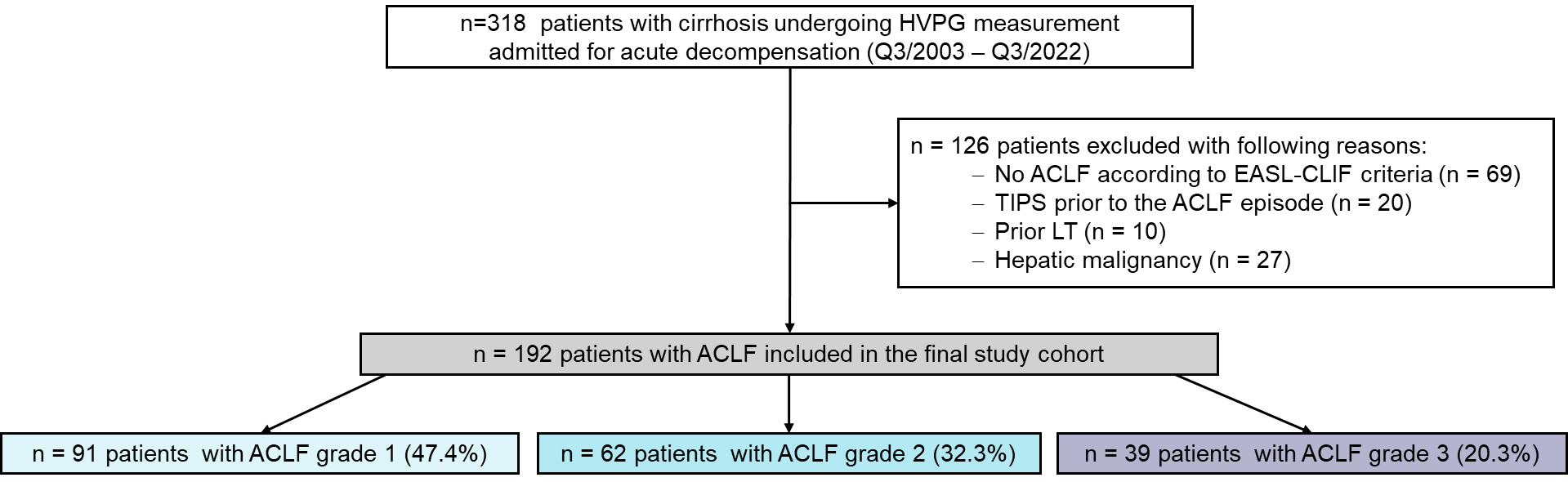


Abbreviations: ACLF, acute-on-chronic liver failure; HVPG, hepatic venous pressure gradient; LT, orthotopic liver transplantation; TIPS, transjugular intrahepatic porto-systemic shunt.

**Supplementary Figure-S2. PH surrogates prior to ACLF stratified according to NSBB treatment status at ACLF diagnosis.**

**
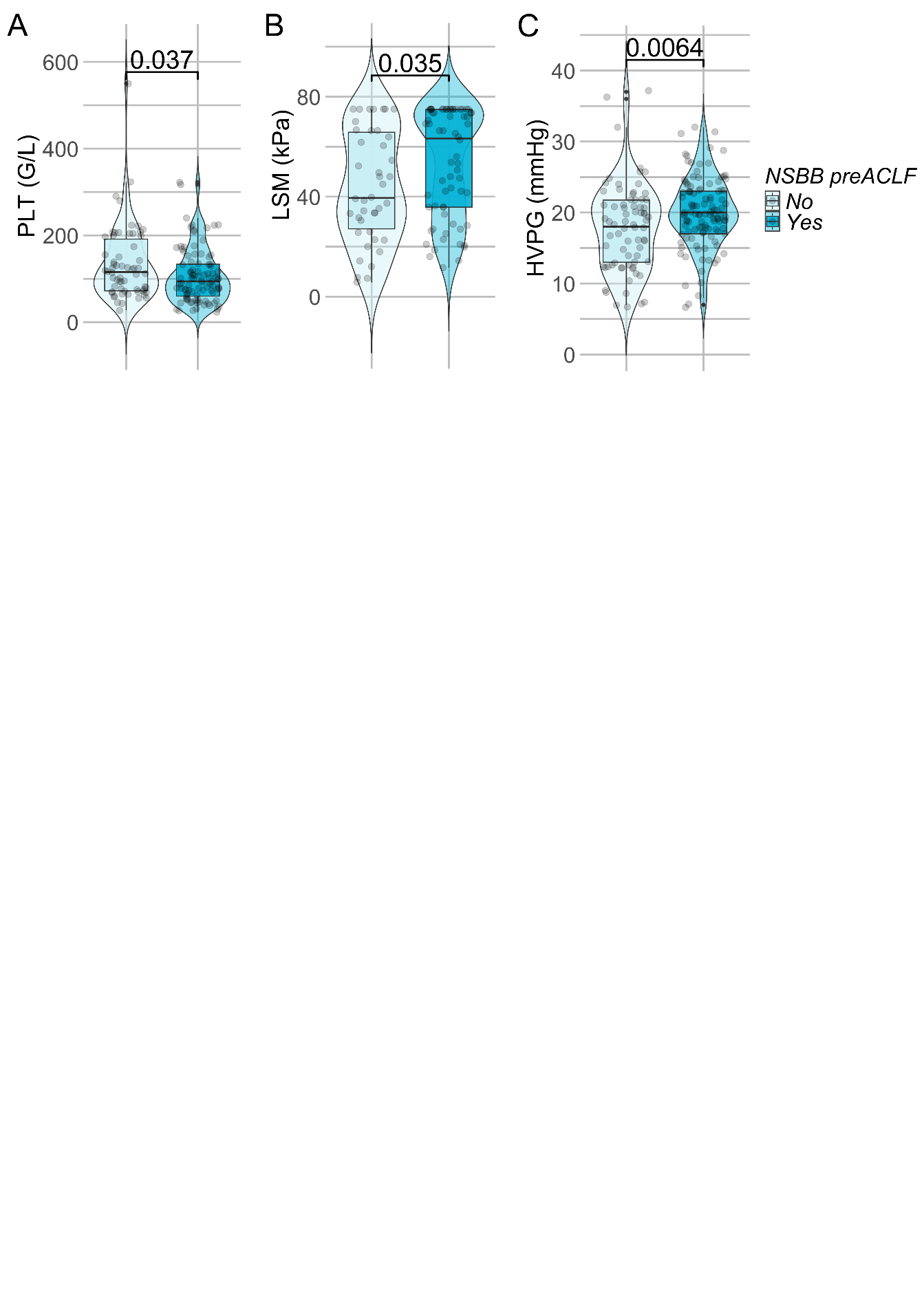
**

Figure legend: A – PLT prior to ACLF; B – LSM prior to ACLF; C – HVPG prior to ACLF. Statistical analysis: continuous variables were compared using Mann-Whitney U test. Abbreviations: ACLF, acute-on-chronic liver failure; HVPG, hepatic venous pressure gradient; LSM, liver stiffness measurement; NSBB, non-selective beta-blocker; PLT, platelet count.

**Supplementary Figure-S3. Change of PH and SI surrogates between D0 and D7 across different ACLF grades.**


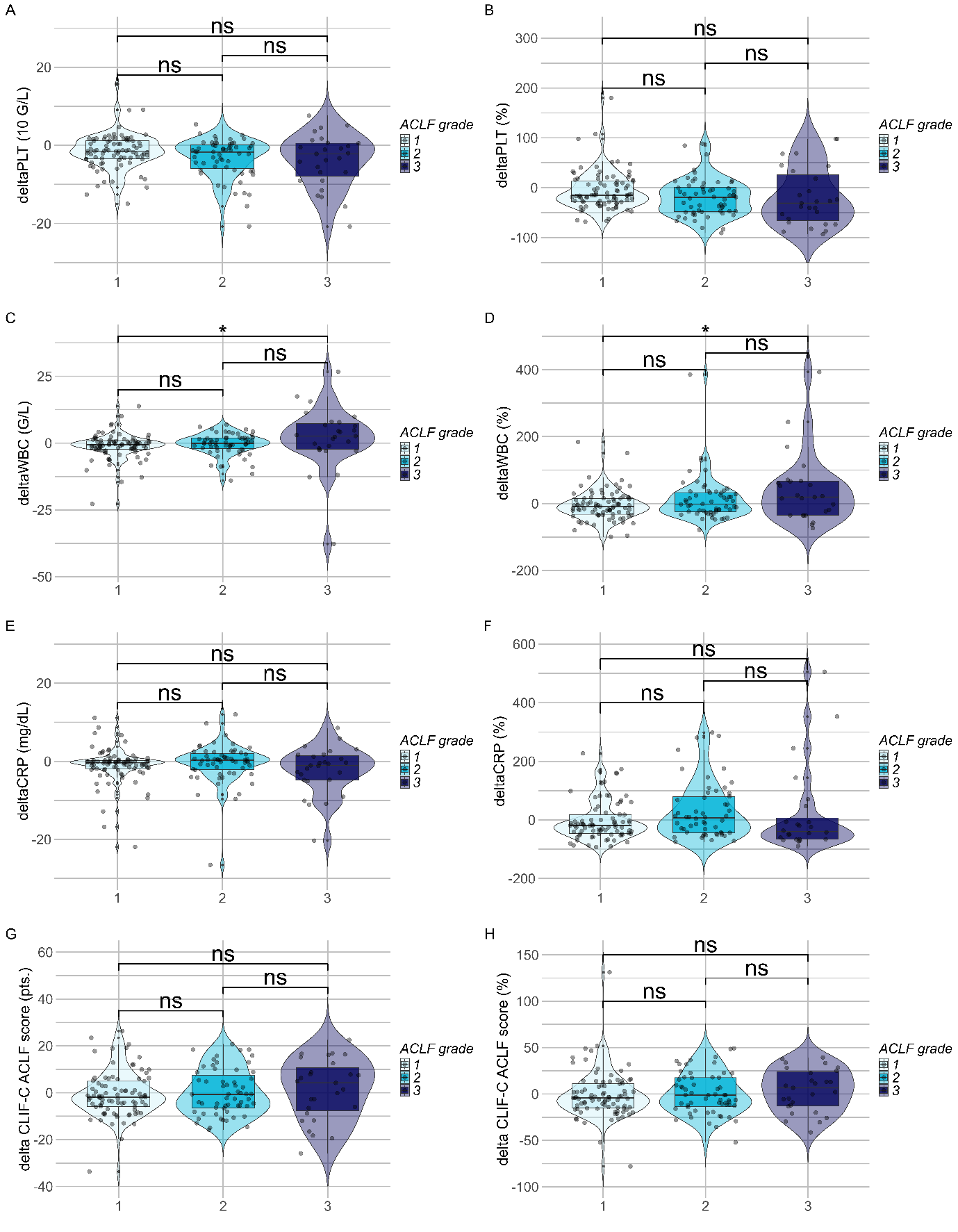


Figure legend: ns, not significant; * p<0.05; ** p<0.01; *** p<0.001. Statistical analysis: Kruskal-Wallis test was used for multiple-groups comparisons of metric variables and adjusted for multiple testing using Dunn’s correction. Abbreviations: ACLF, acute-on-chronic liver failure; CLIF-C, chronic liver failure consortium; CRP, C reactive protein; PLT-platelet count; WBC, white blood cell count.

**Supplementary Figure-S4. Prior NSBB treatment impact on severity and outcome of ACLF.**


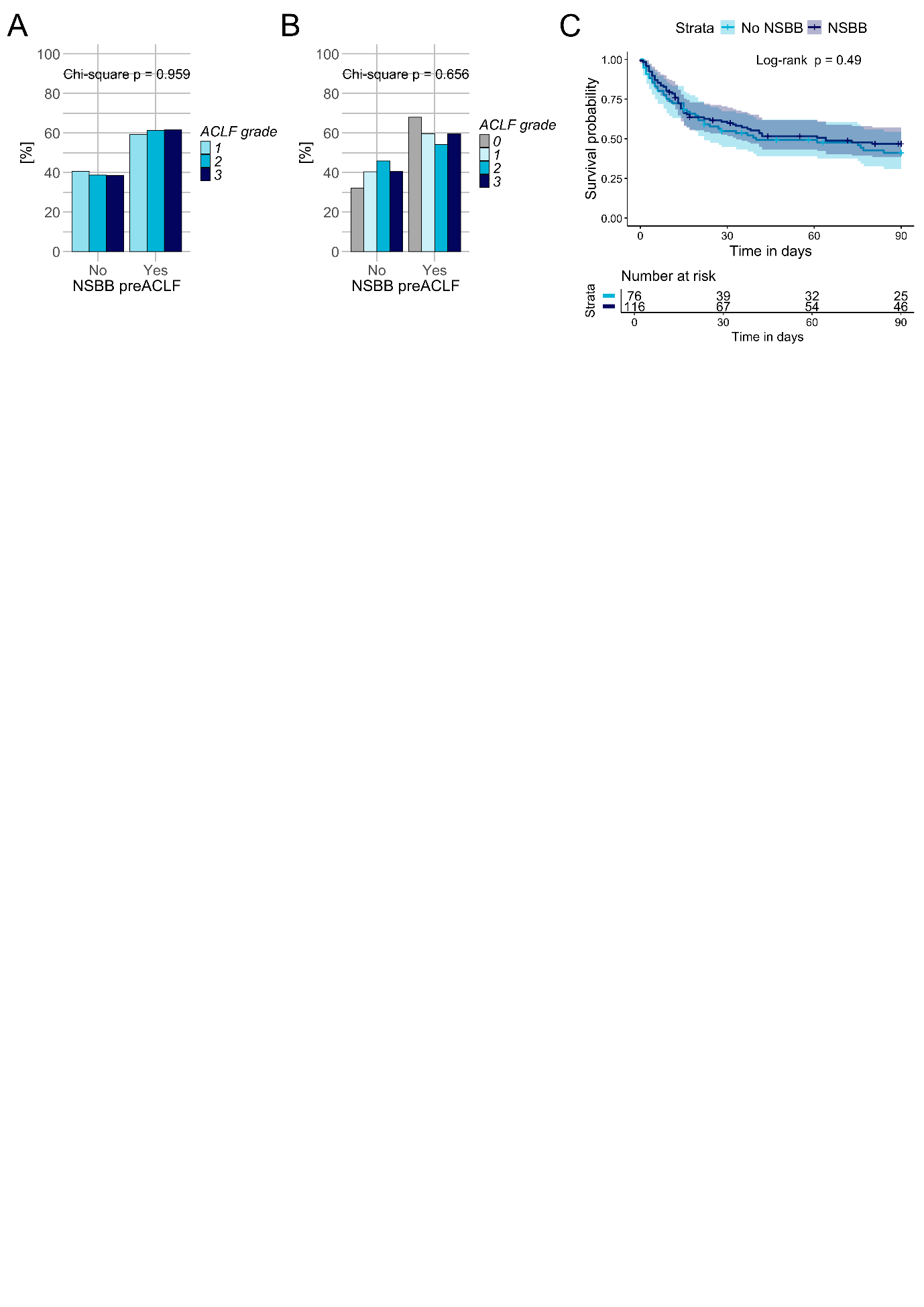


Figure legend: A – distribution of ACLF grades at D0 stratified by NSBB status pre-ACLF; B – distribution of ACLF grades at D7 stratified by NSBB status pre-ACLF; C – day 90 survival stratified by NSBB status pre-ACLF. Statistical analysis: categorical variables were compared using Chi-squared tests. Survival probabilities were displayed using Kaplan-Meier curves and compared using log-rank test. Abbreviations: ACLF, acute-on-chronic liver failure; NSBB, non-selective beta-blocker.

**Supplementary Figure-S5. PLT differences in patients with/without confirmed infection at D0 and D7.**


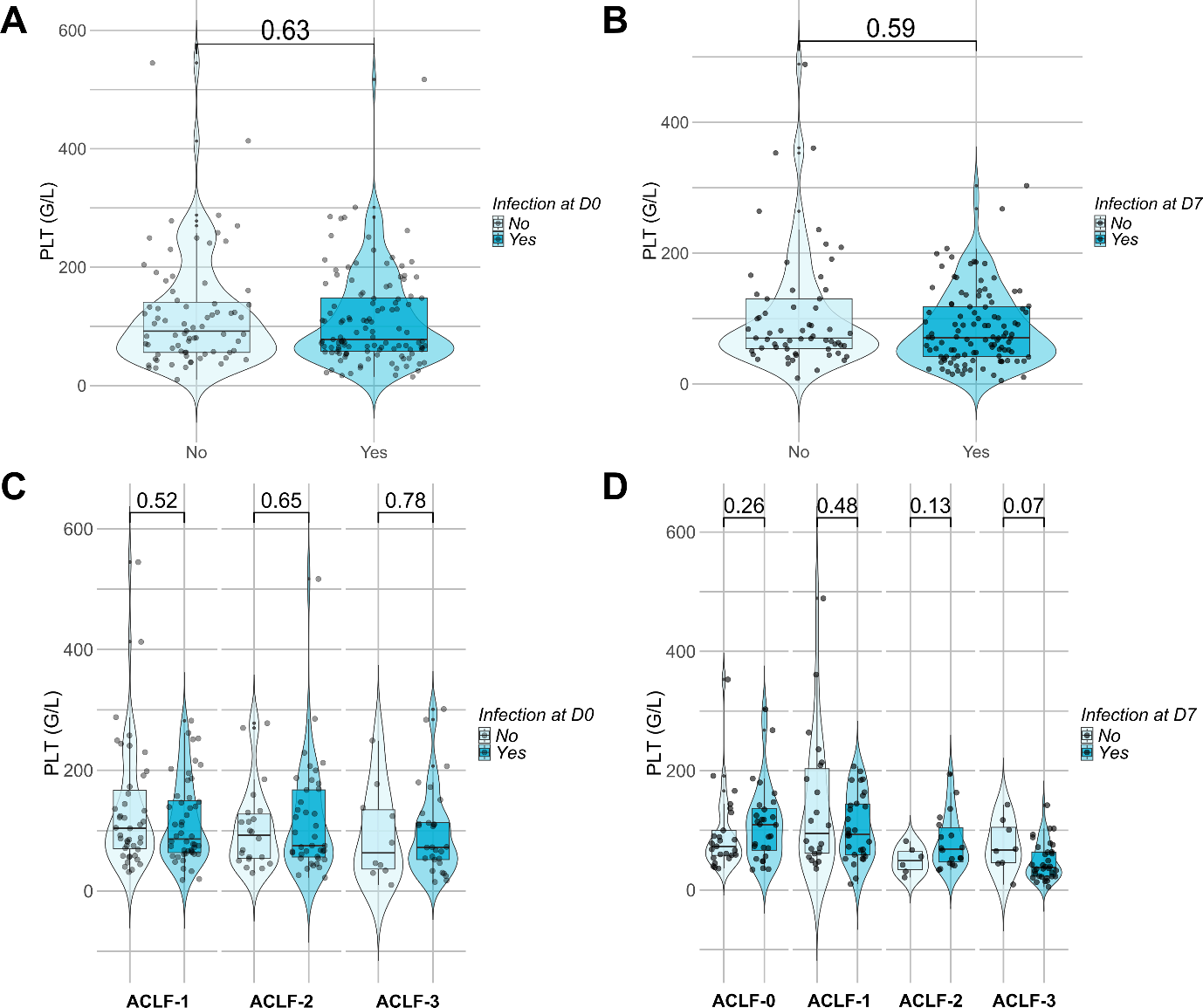


Figure legend: A – comparison of PLT count between patients with/without confirmed infection at D0; B – comparison of PLT count between patients with/without confirmed infection at D7; C – comparison of PLT count between patients with/without confirmed infection stratified by ACLF grade at D0; D – comparison of PLT count between patients with/without confirmed infection stratified by ACLF grade at D7. Statistical analysis: Mann-Whitney U test was used to compare metric variables between groups. Abbreviations: ACLF, acute-on-chronic liver failure, PLT, platelet count.

**Supplementary Figure-S6. Relationship between dynamics of thrombocytopenia and systemic inflammation / coagulopathy during ACLF.**

**
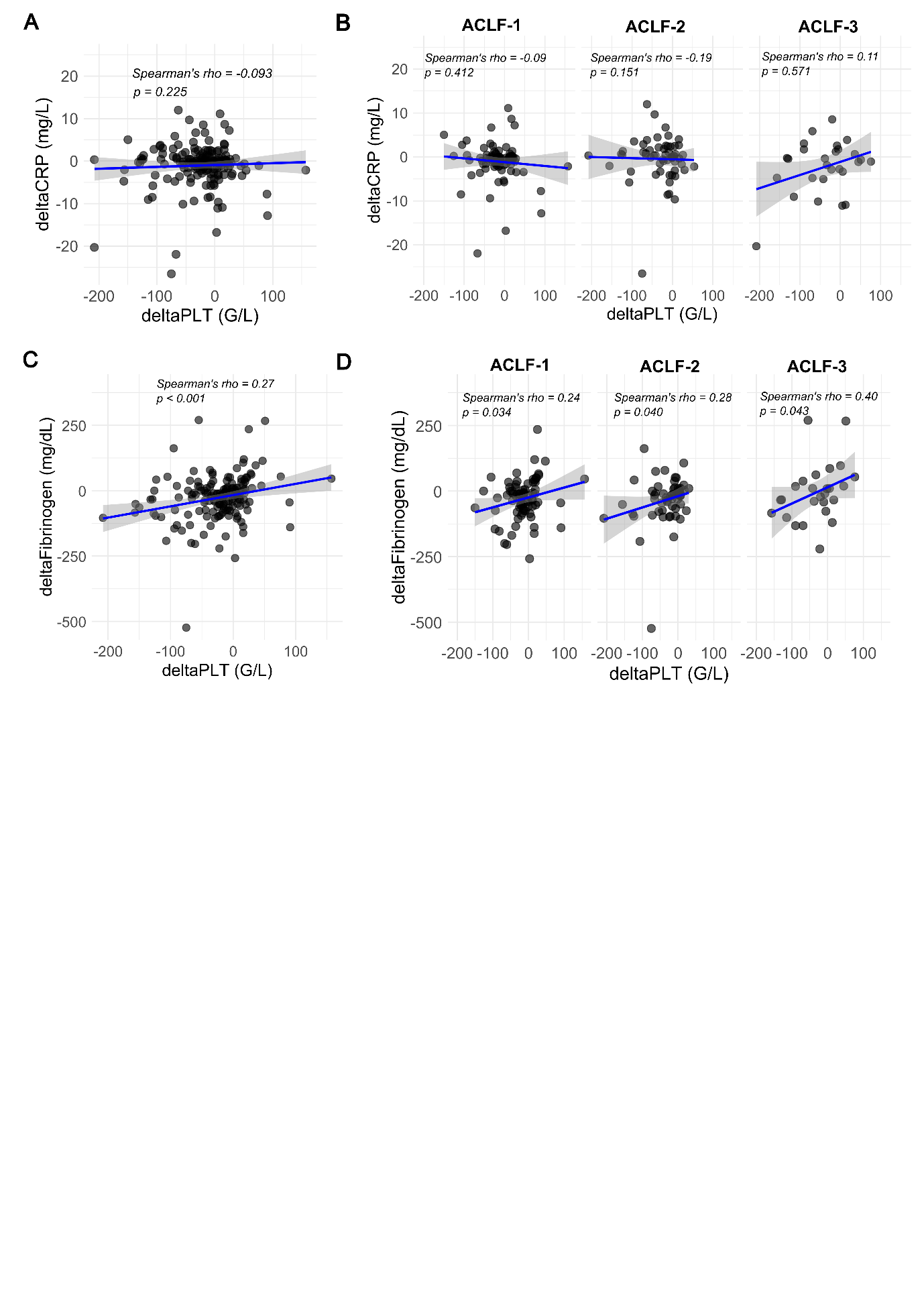
**

Figure legend: A – correlation between deltaPLT and deltaCRP in the overall cohort; B – correlation between deltaPLT and deltaCRP in patients stratified by ACLF grade at D0; C – correlation between deltaPLT and deltaFibrinogen in the overall cohort; D – correlation between deltaPLT and deltaFibrinogen in patients stratified by ACLF grade at D0. Statistical analysis: Spearman’s rho correlation was used to analyze the association between metric variables. Abbreviations: deltaCRP, C-reactive protein change between D0 and D7; deltaFibrinogen, fibrinogen change between D0 and D7; deltaPLT, platelet count change between D0 and D7.

**Supplementary Figure-S7. Change of fibrinolysis surrogates between D0 and D7 across different ACLF grades.**


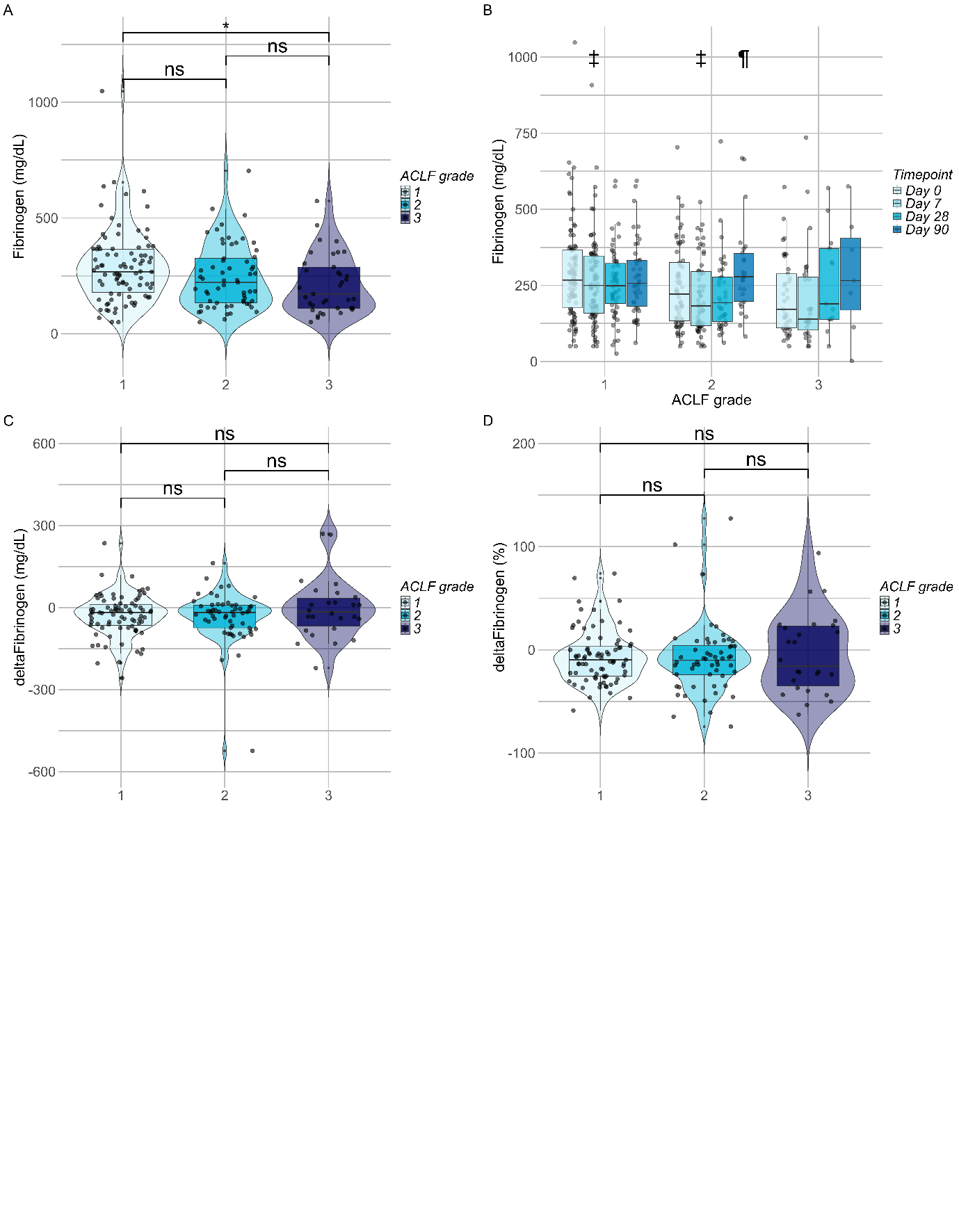


Figure legend: A – comparison of blood fibrinogen levels between different ACLF grades at D0; B – dynamics of blood fibrinogen levels during and after ACLF; C – comparison of absolute changes in blood fibrinogen from D0 to D7 in patients stratified on ACLF grade at D0; D - comparison of relative changes in blood fibrinogen from D0 to D7 in patients stratified on ACLF grade at D0. Statistical analysis: Kruskal-Wallis test was used for multiple-groups comparisons of metric variables and adjusted for multiple testing using Dunn’s correction (ns, not significant; * p<0.05); paired Wilcoxon signed-rank test was applied to compare fibrinogen levels at different timepoints (‡ p<0.05 compared to D0; § p<0.05 compared to D7; ¶ p<0.05 compared to D28).

**Supplementary Figure-S8. Effects of continuous covariates of the prediction model on D28 mortality.**


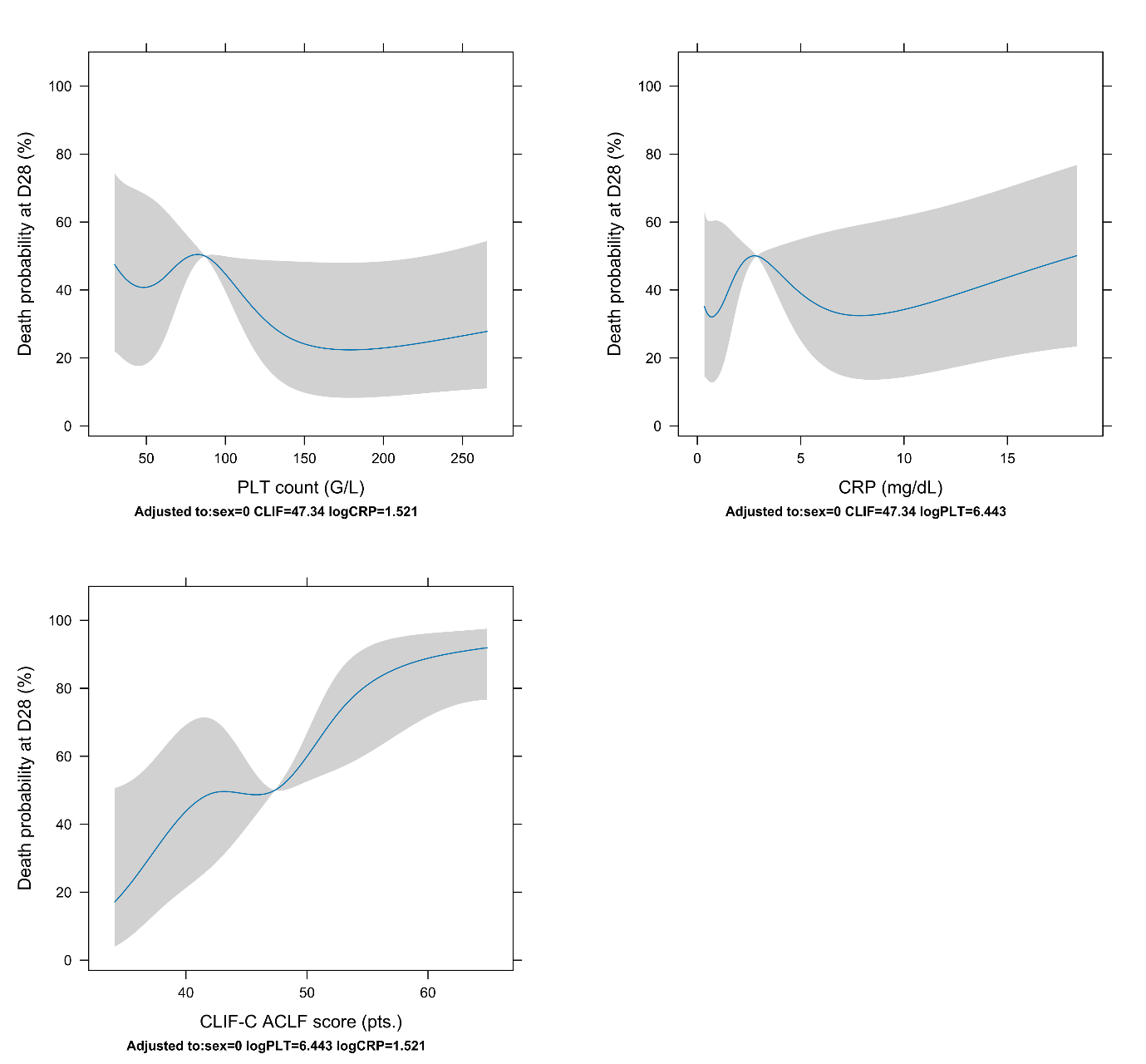


Statistical analysis: the effect of each metric covariate on the outcome was varied, while adjusting for the other covariates in the model. Abbreviations: CLIF-C ACLF, chronic liver failure consortium acute-on-chronic liver failure score; CRP, C reactive protein; PLT, platelet.

**Supplementary Figure-S9. Apparent performance and calibration of the prediction model.**


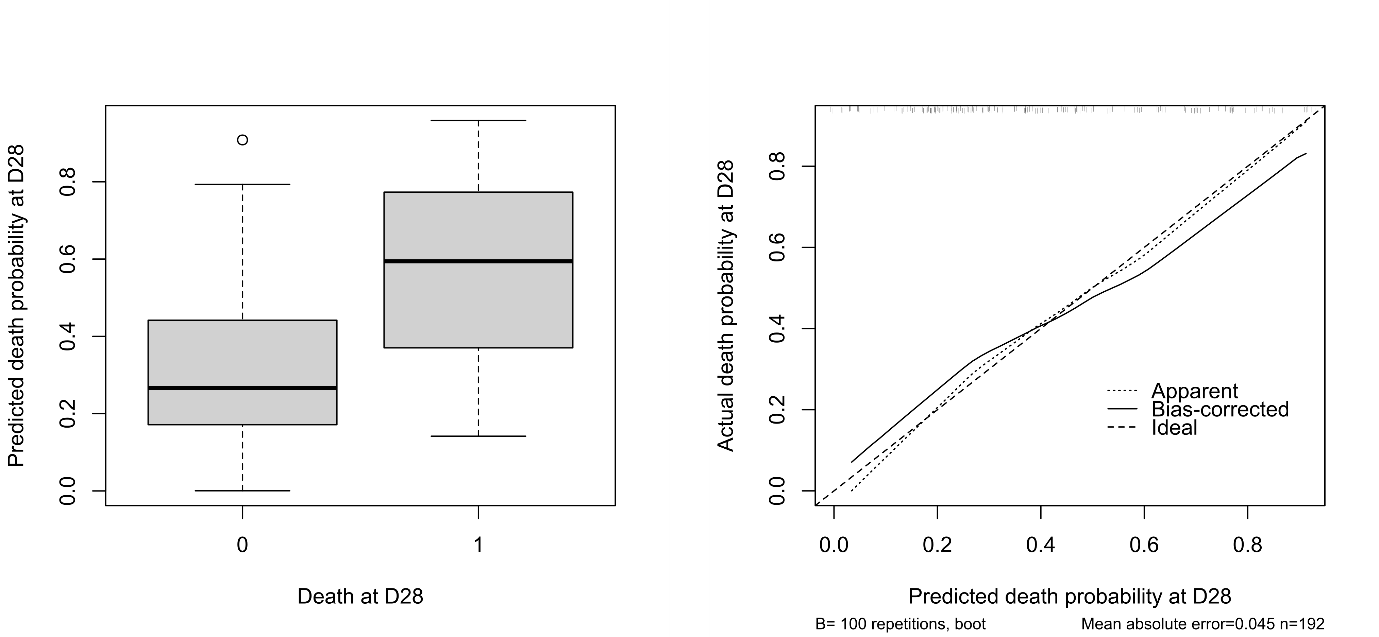


Statistical analysis: apparent performance (left) was assessed by plotting the model predicted probabilities against the actual outcome. Model calibration was visually assessed (right) by plotting the apparent calibration, as well as the bootstrap bias-corrected slopes.

**Supplementary Figure-S10. Comparison of the of established prediction scores and the model including PLT.**


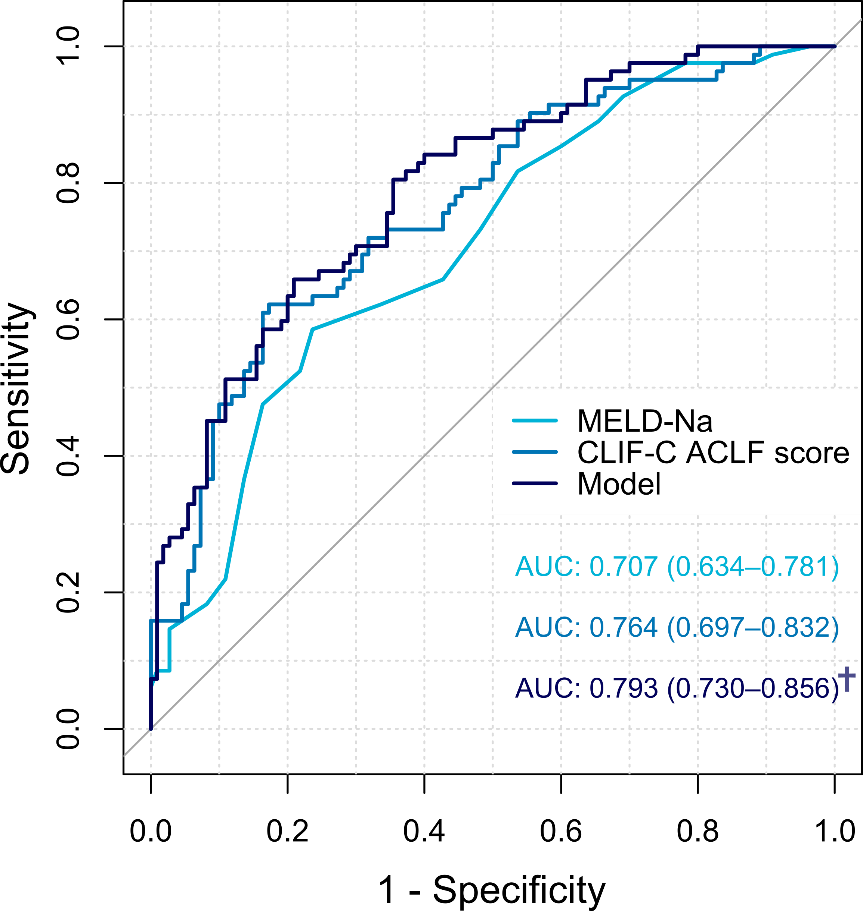


Figure legend: † p<0.05 compared to MELD-Na score; ‡ p<0.05 compared to CLIF-C ACLF score. Statistical analysis: AUROC analysis was performed to assess the discriminating performance of the different predicting variables, while DeLong’s test was used to compare their performances. Abbreviations: CLIF-C ACLF, chronic liver failure consortium acute-on-chronic liver failure score; MELD, model for end-stage liver disease.

# **Supplementary tables**

**Supplementary Table-S1. Missing values of the key parameters at different study timepoints.**

| **Variable** | **preACLF** | **D0** | **D7** | **D28** | **D90** |
| --- | --- | --- | --- | --- | --- |
| Patients (alive) (n) | **192** | **192** | **172** | **115** | **89** |
| Varices (n, %) | 17 (9) | - | - | - |  |
| Splenomegaly (n, %) | 20 (10) | - | - | - |  |
| HVPG ≤1y pre ACLF (n, %) | 95 (51) | - | - | - |  |
| PLT count (n, %) | 35 (18) | 0 (0) | 1 (0.5) | 3 (2.6) | 11 (12.3) |
| VWF (n, %) | 101 (53) | 162 (84) | 163 (95) | 96 (83) | 60 (71) |
| WBC (n, %) | 35 (18) | 0 (0) | 1 (0.5) | 3 (2.6) | 11 (12.3) |
| CRP (n, %) | 35 (18) | 0 (0) | 1 (0.5) | 3 (2.6) | 11 (12.3) |
| IL-6 (n, %) | 167 (87) | 179 (93) | 158 (92) | 108 (94) | 74 (88) |
| Ammonia (n, %) | 84 (44) | 50 (26) | 69 (40) | 48 (42) | 23 (27) |
| MELD-Na (n, %) | 35 (18) | 0 (0) | 1 (0.5) | 3 (2.6) | 11 (12.3) |
| CLIF-C ACLF (n, %) | - | 0 (0) | 1 (0.5) | - | - |

Abbreviations: ACLF, acute-on-chronic liver failure; CLIF-C, chronic liver failure consortium; CRP, C-reactive protein; HVPG, hepatic venous pressure gradient; IL-6, interleukin 6; LSM, liver stiffness measurement; MELD-Na, model for end-stage liver disease including sodium; PLT, platelet count; VWF, von Willebrand factor antigen; WBC, white blood cells.

**Supplementary Table-S2. Characteristics of patients with available HVPG measurement prior to ACLF.**

| **Patient characteristics** | **HVPG low**  **<20 mmHg** | **HVPG high**  **≥20 mmHg** | **P value** |
| --- | --- | --- | --- |
| Patients (n, %) | 39 (41) | 56 (59) | - |
| Age (years) | 55.6 ± 13.1 | 56.8 ± 10.7 | 0.623 |
| Sex (male; n, %) | 22 (56) | 40 (71) | 0.130 |
| Etiology (n, %) |  |  | 0.123 |
| ALD | 19 (49) | 35 (63) |  |
| MASLD | 4 (10) | 4 (7) |  |
| VIRAL | 7 (18) | 12 (21) |  |
| CHOL | 5 (13) | 5 (9) |  |
| OTHER | 4 (10) | 0 (0) |  |
| **Pre-ACLF status** | | | |
| Varices (n, % of known) | 20 (65) | 44 (83) | 0.055 |
| Splenomegaly (n, % of known) | 24 (67) | 37 (79) | 0.217 |
| Ascites (n, % of known) | 27 (73) | 47 (87) | 0.091 |
| LSM (kPa) | 47.4 (25.8) | 74.6 (24.9)^†^ | **0.002** |
| HVPG (mmHg) | 15.0 (6.0) | 23.0 (5.0)^†^ | **<0.001** |
| PLT (G/L) | 119 (111) | 110 (68) | 0.246 |
| VWF (%) | 382 (127) | 413 (115) | 0.157 |
| WBC (G/L) | 6.2 (5.4) | 6.3 (4.4) | 0.921 |
| CRP (mg/dL) | 1.2 (3.1) | 1.5 (2.9) | 0.771 |
| Ammonia (µmol/L) | 35.0 (36.4) | 51.6 (24.5) | 0.111 |
| Child-Pugh class (A/B/C) | 2/14/15 | 1/26/22 | 0.529 |
| MELD-Na score | 19 ± 6 | 20 ± 6 | 0.813 |
| **At ACLF diagnosis (D0)** | | | |
| GI bleeding (n, %) | 4 (10) | 10 (18) | 0.304 |
| Infection (n, %) | 22 (56) | 27 (48) | 0.432 |
| Creatinine (mg/dL) | 2.2 (1.4) | 2.1 (1.0) | 0.880 |
| PLT (G/L) | 119 (114) | 83 (73) | 0.057 |
| VWF (%) | 413 (117) | 420 (34) | 0.927 |
| WBC (G/L) | 8.7 (6.9) | 8.0 (5.7) | 0.258 |
| CRP (mg/dL) | 2.2 (3.9) | 2.5 (5.0) | 0.518 |
| Ammonia (µmol/L) | 77.8 (73.7) | 65.3 (67.1) | 0.826 |
| MELD-Na score | 27 ± 6 | 27 ± 6 | 0.913 |
| CLIF-C OF score | 10 (3) | 9 (2) | 0.526 |
| Liver failure (no/dysfunction/failure) | 22/6/11 | 36/8/12 | 0.709 |
| Kidney failure (no/dysfunction/failure) | 12/19/8 | 21/29/6 | 0.398 |
| Circulatory failure (no/dysfunction/failure) | 31/1/7 | 37/5/14 | 0.277 |
| Respiratory failure (no/dysfunction/failure) | 35/1/3 | 48/1/7 | 0.736 |
| Brain failure (no/dysfunction/failure) | 15/7/17 | 24/12/20 | 0.736 |
| Coagulation failure (no/dysfunction/failure) | 25/9/5 | 38/8/10 | 0.494 |
| ACLF grade (1/2/3) | 18/14/7 | 31/17/8 | 0.674 |
| CLIF-C ACLF score | 48.2 ± 8.7 | 46.0 ± 11.2 | 0.321 |
| CLIF-C AD score | 62.9 ± 11.1 | 61.2 ± 13.4 | 0.510 |
| **At D7** | | | |
| GI bleed (n, %) | 3 (9) | 4 (8) | 0.851 |
| Infection (n, %) | 20 (59) | 32 (62) | 0.801 |
| Creatinine (mg/dL) | 1.4 (1.5) | 1.6 (1.7) | 0.262 |
| PLT (G/L) | 109 (119) | 67 (79)^†^ | **0.014** |
| WBC (G/L) | 7.5 (5.6) | 7.6 (8.1) | 0.943 |
| CRP (mg/dL) | 1.9 (3.5) | 2.8 (3.6) | 0.083 |
| Ammonia (µmol/L) | 59.2 (60.9) | 54.2 (45.9) | 0.706 |
| MELD-Na score | 25 ± 7 | 26 ± 8 | 0.360 |
| CLIF-C OF score | 9 (3) | 9 (5) | 0.772 |
| Liver failure (no/dysfunction/failure) | 16/10/8 | 32/9/11 | 0.335 |
| Kidney failure (no/dysfunction/failure) | 17/12/5 | 30/14/8 | 0.703 |
| Circulatory failure (no/dysfunction/failure) | 29/0/5 | 34/1/17 | 0.113 |
| Respiratory failure (no/dysfunction/failure) | 32/1/1 | 41/3/8 | 0.138 |
| Brain failure (no/dysfunction/failure) | 19/7/8 | 25/13/14 | 0.775 |
| Coagulation failure (no/dysfunction/failure) | 23/7/4 | 35/6/11 | 0.341 |
| ACLF grade (0/1/2/3) | 6/18/7/3 | 16/15/7/13^†^ | **0.046** |
| CLIF-C ACLF score | 43.7 ± 9.0 | 45.6 ± 15.9 | 0.499 |
| CLIF-C AD score | 56.4 ± 10.5 | 58.5 ± 15.7 | 0.493 |
| **At D28** | | | |
| MELD-Na score | 24 ± 7 | 21 ± 7 | 0.228 |
| CLIF-C AD score | 55.4 (10.4) | 52.1 (10.7) | 0.117 |
| **At D90** | | | |
| MELD-Na score | 18 (8) | 19.5 (8) | 0.812 |
| CLIF-C AD score | 53.2 (11.4) | 52.9 (10.5) | 0.747 |
| **Liver-related death post-ACLF (n, %)** | | | |
| In hospital mortality | 15 (39) | 26 (46) | 0.276 |
| D7 mortality | 7 (18) | 10 (18) | 0.918 |
| D28 mortality | 13 (34) | 24 (43) | 0.506 |
| D90 mortality | 16 (43) | 29 (52) | 0.468 |
| Time from HVPG to D0  (median in days, IQR) | 29 (203) | 38 (169) | 0.823 |
| Transplant-free survival  (median in months, 95% CI)^¶^ | 6.6 (1.3-50.1) | 2.5 (0.7-16.9)^†^ | **0.035** |

Statistical analysis: metric variables are presented as median (IQR) or mean ± SD, depending on the normal distribution of variables. Student T tests and Mann-Whitney U tests were used to compare parametric and non-parametric variables, respectively.. Survival rates were compared using log-rank tests. † p<0.05 when compared to HVPG low. Abbreviations: ACLF, acute-on-chronic liver failure; AD, acute decompensation; CLIF-C, chronic liver failure consortium; CRP, C-reactive protein; MELD-Na, model for end-stage liver disease including sodium; OF, organ failure; PLT, platelet count; VWF, von Willebrand factor antigen; WBC, white blood cells.

**Supplementary Table-S3. Univariable Cox-regression using D0 platelet count to predict day 7, day 28, day 90 mortality.**

| **At D0** | **D7 mortality**  HR [95% C.I.] | **D28 mortality**  HR [95% C.I.] | **D90 mortality**  HR [95% C.I.] |
| --- | --- | --- | --- |
| PLT (per 10 G/L) | 0.98  [0.94-1.03] | 0.96  [0.93-0.99] | 0.97  [0.94-0.99] |
| P value | 0.504 | **0.027** | **0.021** |

Statistical analysis: univariable Cox proportional hazard models were used to determine the prognostic value of metric variables towards mortality risk at different timepoints. Abbreviations: C.I., confidence interval; HR, hazard ratio; PLT, platelet count.

**Supplementary Table-S4. Predictors of ACLF D28 mortality assessed at D7.**

| **Covariates** | **Univariable analysis** | | | **Multivariable analysis (last step)** | | |
| --- | --- | --- | --- | --- | --- | --- |
|  | **HR** | **95% C.I.** | **P value** | **aHR** | **95% C.I.** | **P value** |
| PLT (per 10 G/L) | 0.86 | 0.80-0.93 | **<0.001** |  |  |  |
| deltaPLT (per 10 G/L) | 0.94 | 0.90-0.99 | **0.014** | 0.99 | 0.98-0.99 | **<0.001** |
| deltaPLT (per %) | 0.99 | 0.98-0.99 | **<0.001** |  |  |  |
| WBC (per G/L) | 1.10 | 1.07-1.14 | **<0.001** |  |  |  |
| deltaWBC (per G/L) | 1.08 | 1.02-1.13 | **0.004** |  |  |  |
| deltaWBC (per %) | 1.00 | 1.00-1.00 | 0.697 |  |  |  |
| CRP (per mg/dL) | 1.09 | 1.02-1.15 | **0.007** | 1.04 | 0.99-1.11 | 0.205 |
| deltaCRP (per mg/dL.) | 1.00 | 0.95-1.06 | 0.939 |  |  |  |
| deltaCRP (per %) | 1.00 | 1.00-1.00 | 0.710 |  |  |  |
| MELD-Na score (per point) | 1.17 | 1.12-1.22 | **<0.001** |  |  |  |
| CLIF-C AD score (per point) | 1.11 | 1.08-1.14 | **<0.001** |  |  |  |
| CLIF-C ACLF score  (per point) | 1.12 | 1.09-1.14 | **<0.001** |  |  |  |
| deltaCLIF-C ACLF score (per point) | 1.15 | 1.11-1.18 | **<0.001** | 1.15 | 1.11-1.18 | **<0.001** |
| deltaCLIF-C ACLF score (per %) | 1.02 | 1.01-1.03 | **<0.001** |  |  |  |

Statistical analysis: multivariable analysis was performed using a backward stepwise Cox proportional hazards regression model. Abbreviations: CLIF-C AD / ACLF, chronic liver failure acute decompensation / acute-on-chronic liver failure score; CRP, C reactive protein; HR, hazard ratio; MELD, model for end-stage liver disease; PLT, platelet count; WBC, white blood cell count.

**Supplementary Table-S5. Diagnostics of the prediction model including CLIF-C ACLF and PLT at D0.**

| **Parameters** | **Original cohort** | **Training set** | **Test set** | **Corrected** | **Bootstrap (n)** |
| --- | --- | --- | --- | --- | --- |
| **C-index** | 0.79 | 0.82 | 0.77 | 0.74 | 100 |
| **R-squared** | 0.34 | 0.40 | 0.29 | 0.22 | 100 |
| **Intercept** | 0.00 | 0.00 | -0.07 | -0.07 | 100 |
| **Slope** | 1.00 | 1.00 | 0.73 | 0.73 | 100 |
| **Emax** | 0.00 | 0.00 | 0.08 | 0.08 | 100 |
| **Discrimination** | 0.29 | 0.36 | 0.23 | 0.17 | 100 |
| **Brier score** | 0.18 | 0.17 | 0.20 | 0.21 | 100 |

Statistical analysis: a multivariable logistic regression model fitted with natural splines with 3 degrees of freedom for continuous variables was used. The table displays the diagnostics of the bootstrap (n=100) validation of the model.
